# Insight into biases and sequencing errors for amplicon sequencing with the Illumina MiSeq platform

**DOI:** 10.1093/nar/gku1341

**Published:** 2015-01-13

**Authors:** Melanie Schirmer, Umer Z. Ijaz, Rosalinda D'Amore, Neil Hall, William T. Sloan, Christopher Quince

**Affiliations:** 1 School of Engineering, University of Glasgow, Glasgow, UK; 2 Functional and Comparative Genomics, University of Liverpool, Liverpool, UK

## Abstract

With read lengths of currently up to 2 × 300 bp, high throughput and low sequencing costs Illumina's MiSeq is becoming one of the most utilized sequencing platforms worldwide. The platform is manageable and affordable even for smaller labs. This enables quick turnaround on a broad range of applications such as targeted gene sequencing, metagenomics, small genome sequencing and clinical molecular diagnostics. However, Illumina error profiles are still poorly understood and programs are therefore not designed for the idiosyncrasies of Illumina data. A better knowledge of the error patterns is essential for sequence analysis and vital if we are to draw valid conclusions. Studying true genetic variation in a population sample is fundamental for understanding diseases, evolution and origin. We conducted a large study on the error patterns for the MiSeq based on 16S rRNA amplicon sequencing data. We tested state-of-the-art library preparation methods for amplicon sequencing and showed that the library preparation method and the choice of primers are the most significant sources of bias and cause distinct error patterns. Furthermore we tested the efficiency of various error correction strategies and identified quality trimming (Sickle) combined with error correction (BayesHammer) followed by read overlapping (PANDAseq) as the most successful approach, reducing substitution error rates on average by 93%.

## INTRODUCTION

The announcement by Roche to withdraw the GS FLX 454 pyrosequencing platform emphasizes the need for a better understanding of Illumina errors. 454 and Illumina sequencing errors are fundamentally different and require different strategies with regard to the downstream analysis. The majority of errors in 454 data are related to homopolymers (1,2). For Illumina, on the other hand, substitution type miscalls are the dominant source of errors. The Illumina sequencing technology is based on array formation. The sequencing templates are immobilized on a flow cell and a subsequent solid-phase bridge amplification generates up to 1000 copies in close proximity (cluster generation). The sequencing-by-synthesis technology uses fluorescently labelled reversible terminator-bound dNTPs (deoxyribonucleotides: A,C,G,T) for the polymerization. Only one base is added in each step due to the 3′ termination of the incorporated nucleotide. The fluorophores are illuminated by a red laser for A and C and a green laser for G and T and imaged through different filters to identify the four different nucleotides. The fluorescent labels and the 3′ terminators are then removed in order for the next cycle to commence. Challenges arise due to a strong correlation of A and C as well as G and T intensities as a result of similar emission spectra of the fluorophores and limitations of the filters that are used to separate the signals. Furthermore problems known as phasing and pre-phasing can cause noise in the cluster signal. Phasing can occur due to problems with the enzyme kinetics such as incomplete removal of the 3′ terminators or the fluorophores and cause the synthesis of some molecules in a cluster to lag behind. During pre-phasing on the other hand the synthesis advances too fast which can be caused by inadequate flushing of the flow cell, by sequences in a cluster skipping an incorporation cycle or the incorporation of nucleotides without an effective 3′ terminator. The number of affected sequences increases with each cycle and thus limits the overall read length. Overall, substitution type miscalls are the major source of errors for Illumina sequencing (3).

Most previous studies on Illumina-specific errors have concentrated on the Genome Analyzer (GAII) and the HiSeq 2000 (4–6). Significant improvements in the technology and software have generally improved error rates but we still face systematic errors in Illumina sequencing. Nakamura *et al*. (7) identified two sequence patterns in Illumina GAII data that trigger errors during the sequencing process—firstly, inverted repeats and secondly, GGC sequences. They suspect that the first pattern causes dephasing by inhibiting single-base elongation through folding of single-stranded DNA and that the second pattern causes altered enzyme preference on the lagging-strand. They also report that mismatches are mainly observed in reads sequenced in the same direction and there is a strong correlation between average base call quality and mismatch rate. Their study also showed that the GGC pattern does not always trigger a sequencing error. The pattern ‘may occur once every 64 bases by chance’ (7) but sequence-specific errors (SSEs) are less common. Furthermore a significant number of SSE positions were not associated with the identified sequence patterns suggesting that other factors may be a significant cause for sequencing errors.

For our experiments we used a variety of single species samples as well as a complex mock community consisting of 59 organisms. We developed a program that enables us to infer error profiles based on sequencing data from mock communities. Erroneous base calls are identified based on the alignment of the reads against the known reference genomes. Our software can identify mismatches, insertions and deletions for any sequenced mock data set. This allowed us to study and compare the impact of: library preparation methods, run, input DNA amount, number of polymerase chain reaction (PCR) cycles, Taq, DNA template and forward and reverse primer combinations. We provide an in-depth analysis of the errors occurring on both read directions for all types of errors and tested the reliability of quality scores. It has been reported previously that the per-base quality scores can be inaccurate and co-variation has been observed with attributes like sequencing technology, machine cycle and sequence context (8). We will show that the accuracy of the quality scores varies depending on which library preparation method was used. The differentiation of true variation and context-specific sequencing errors is a major challenge in next generation sequencing (NGS) analysis. Being able to infer error profiles for individual sequencing runs has the potential to greatly improve our ability to correct errors and thus enhance further sequencing analysis.

Currently available programs aiming to address errors in Illumina sequencing data include quality trimming, error correction and read overlapping. During quality trimming the average quality scores are computed over a sliding window across the whole read. The start of the read is trimmed until the average quality score is larger than a certain threshold and the end of the read is trimmed if the average quality score falls below the threshold. Error correction will be tested with the program BayesHammer. During this approach a Hamming graph is constructed based on the k-mer composition of the reads. Using Bayesian subclustering the connected components are further divided into subclusters taking the quality values into account. For each subcluster the central k-mer is assumed to be the true sequence and the remaining sequences are corrected accordingly. Errors can also be corrected by overlapping paired-end reads. The reads are aligned and the optimal overlap is determined followed by error correction and assembling of the reads into a single sequence. Here, the quality scores are used for aligning the reads as well as for the error correction. We will test the capacity of these approaches and discuss their limitations.

## MATERIALS AND METHODS

### Mock community and sequencing data

We sequenced a variety of samples ranging from single species to diverse mock communities with different abundance distributions. The single organisms included *Anaerocellum thermophilum Z-1320 DSM6725* (AT), *Bacteroides thetaiotaomicron VPI-5482* (BT), *Bacteroides vulgatus ATCC 8482* (BV), *Herpetosiphon aurantiacus ATCC 23779* (HA), *Rhodopirellula baltica SH 1* (RBS), *Leptothrix cholodnii SP-6* (LC) and *Caldicellulosiruptor saccharolyticus DSM 8903* (CS). For the first mock community we combined even amounts of purified genomic DNA (9) from 49 bacteria and 10 archaea (see Supplementary Table S6 for details). We used the same genomes to construct an uneven mock community where organisms within the same phyla are distributed according to a log-normal distribution and the different phyla in turn follow a log-normal distribution as well. The bacteria make up a total of 90% of the community and the archaea 10% of the community.

We sequenced the V4 and the V3/V4 region of the respective samples and included two samples for which the whole 16S gene was sequenced. Five different library preparation methods were used including nested single index (SI), nested dual index (DI or 5NDI with five random nucleotides before the primer), NexteraXT (XT) and Fusion Golay (FG). (For further details on the library preparation methods see the Supplementary material.) The samples were distributed across seven runs and two MiSeq sequencing machines. We tested a range of different input quantities and tested two DNA polymerases (Kapa HiFi & NEB Q5). In addition we studied the impact of different forward and reverse primer combinations. A detailed list of all data sets including their parameters can be found in the Supplementary material (Supplementary Tables S3 and S4).

### Algorithm for computing the error profiles

First, we aligned the reads with Burrows–Wheeler Aligner (BWA) (Version 0.7.3a-r367) (10) against the reference sequences. Then we converted the alignment to Sequence Alignment/Map (SAM) format using BWA and generated the MD tag with SAMtools (11). Our program then infers position and nucleotide-specific substitution, insertion and deletion rates. The Compact Idiosyncratic Gapped Alignment Report (CIGAR) string encodes matches and mismatches with ‘M’, insertions with an ‘I’ and deletions with ‘D’. Based on the MDtag in the SAM format we then identified the nucleotides that were replaced during a substitution and the types of nucleotides affected during a deletion. From the extended CIGAR string we determined the substituting nucleotides and detected the nucleotides involved in an insertion. In addition we recorded position-specific quality scores for all error types and the 3mers preceding errors (motifs).

Our program outputs 4×*L* matrices for each error type (where *L* is the read length) for the set of R1 and R2 reads, respectively. The number of rows corresponds to the read length and each row specifies the nucleotide-specific error rates for a certain position on the read. The matrices were normalized as follows: firstly, the occurrences of each nucleotide on the read for each position were counted. Secondly, the number of detected substitutions for this nucleotide was added to account for errors where the nucleotide should have been observed as compared to the true reference sequence of the species sample. Lastly, the number of substitutions where the nucleotide was the substituting nucleotide was subtracted, i.e. where a nucleotide was mistaken for another nucleotide. This reflects the true number of occurrences of A, C, G and T.

To verify our algorithm we extended our read simulation program (12) to generate reads based on error profiles of the above-described format. The error profiles, inferred from the simulated reads, concurred with the original error profiles used to simulate the reads. In addition we used mock error profiles with a simple stepwise increase of the error rate along the read for the read simulation. Again, the reconstructed error profiles concurred with the mock profiles used for the simulation and thus validate the algorithm.

### Metric for overall comparison: Hellinger distance

We measured the similarity between the error distributions using the Hellinger distance. The error rates across the read length can be interpreted as probability distributions. We considered substitutions, insertions and deletions separately for R1 and R2 reads, respectively, and summed over the different types of errors in each case.

Definition: Let *P* = (*p*_1_, …, *p*_*L*_) and *Q* = (*q*_1_, …, *q*_*L*_) denote two discrete probability distributions. Then the Hellinger distance *H* is defined as

\begin{equation*} H(P,Q) = \sqrt{\frac{1}{2} \sum _{i=1}^{L} (\sqrt{p_i} - \sqrt{q_i})^2}. \end{equation*}

A value between 0 and 1 is returned. The closer this value is to zero, the more similar the two distributions.

We used a permutation ANOVA (analysis of variance) (http://CRAN.R-project.org/package=vegan) to identify the significant experimental factors for the substitution error patterns as those constitute the majority of errors. We computed the distance matrices using the results based on the Hellinger distance. We chose a model for the permutation ANOVA using the step function in R together with redundancy analysis.

## RESULTS

We only present the detailed results for data set (DS) *DS 35*. However, the same detailed analysis was conducted for all data sets listed in Supplementary Tables S3 and S4. To compare the individual profiles and to identify any patterns associated with particular parameters we then used the Hellinger distance to contrast the error and quality profiles of the different data sets. Subsequently we studied the overall error rates across all library preparation methods and identified associated biases and motifs. We conclude this section by testing the efficiency of several currently available error removal techniques.

### Detailed error and quality profiles for data set *DS 35*

**Figure 1.**
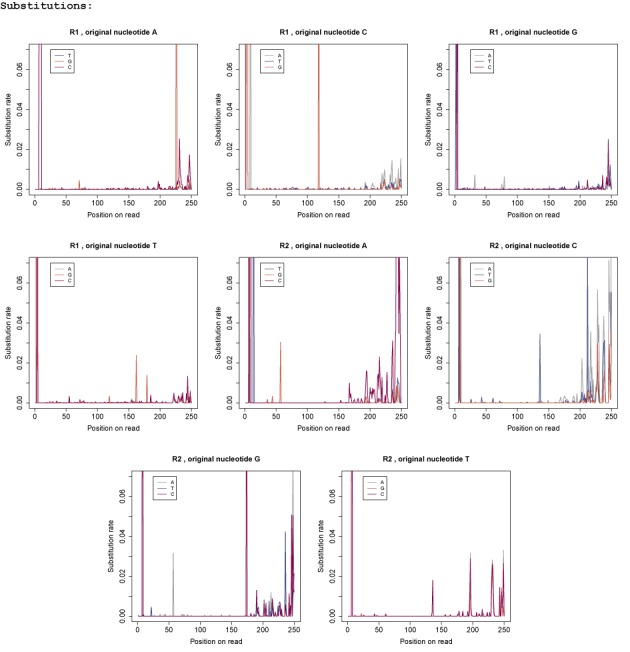
Nucleotide-specific substitution error profiles for data set *DS 35*: each graph shows the substitution rates for a specific original nucleotide and the colours indicate the substituting nucleotide. The first four graphs show the R1 profiles and the last four graphs show the R2 profiles.

For data set *DS 35* the V4 region of the balanced mock community was amplified and the nested DI library preparation method was used. Figure 1 displays the position and nucleotide-specific substitution rates for the R1 and R2 reads, respectively. A small number of errors can result in a high error rate if a nucleotide has very few occurrences at a certain position. In order to avoid overemphasis of these rare errors we smoothed the error profiles for the visualization as follows: for the substitutions we computed the expected minimum number of errors, averaging over all positions. In the case of *DS 35*, T shows the smallest average error rate (0.000262). There are 593,868 R1 reads, so assuming a uniform distribution over nucleotides we would expect ∼148,467 occurrences of each nucleotide at each position and thus ∼38 errors (rounded down). Analogously, we set the minimum threshold for the R2 reads to 144 (smallest nucleotide-specific error rate: 0.000967). The insertion and deletion rates as well as the rates of unknown nucleotides (Ns) were calculated relative to the total coverage of each position. This avoids the problem of overemphasis and hence we do not need to apply a minimum threshold.

#### Substitution error profiles

For all types of substitutions we observed an accumulation of errors across the first 10 bp of the reads. The error rates also increased towards the end of the read in particular for the R2 reads and we can see a clear preference for the substituting nucleotide for some types of substitutions. We compared the substitution preference for each original nucleotide across the last 50 bp. For the R1 reads we detected the following rates: in 66% of the cases A got substituted by C. For C we observed a substitution with A in 58% of the cases. G got substituted by T in 45% of all cases and T got substituted by C in 45% of all cases. For the R2 reads we detected a similar bias: we observed A to C substitutions in 85% of all cases, C to A in 61%, G to T in 40% and T to C in 40% of all cases. We also found that the overall error rate is significantly higher in the R2 reads with ≈0.0107 compared to only ≈0.0064 for the R1 reads.

Other noticeable characteristics were the spikes occurring at certain positions across the reads with error rates much higher than the average error rate. There are several possible underlying reasons for the accumulation of errors at those positions. We first checked if the spikes are likely to be caused by errors in the database. We compared the R2 substitution profiles for three different data sets (see Supplementary Figure S2). In all three cases the organism *B. thetaiotaomicron VPI-5482* was sequenced. For the first data set (column 1) the V4 region of the sample was amplified and prepared with the nested SI, for the second data set (column 2) the V3/V4 region was amplified and the library was constructed with the Nextera XT kit and for the third data set (column 3) the V4 region was amplified and the nested DI was used. For the visualization we smoothed the error profiles accordingly. As all R2 reads cover the V4 region, any issues with the reference database should be visible in all three error profiles. The graphs clearly illustrate that the spikes are not concurrent and thus indicate that it is unlikely that the cause of the spikes are errors in the database. Another indication that the spikes are not a problem with the reference sequences is the rate at which those substitutions occurred. Table 1 gives a selection of spikes that were encountered in *DS 35* specifying the type of substitution, the position and the rate at which the substitution was observed. The organisms in this mock community were initially uniformly distributed. However, PCR amplification introduces a bias as not all sequences are amplified in equal measure. Therefore, we re-calculated the abundance distribution of the 16S rRNA reference genes based on the read alignments. On average each reference sequence accounted for 0.86% of the population with a maximum of 2.8%. Thus the frequency of each 16S rRNA sequence is in most cases significantly lower than the error rate of the respective spike and errors would need to occur simultaneously in multiple sequences to account for the observed rates.

**Table 1.** A selection of substitutions that occurred at a very high rate in data set *DS 35*

R1			R2		
A −> G	pos 226	Rate 0.25	A −> G	pos 57	Rate 0.03
T −> G	pos 162	Rate 0.02	T −> C	pos 136	Rate 0.02
T −> G	pos 179	Rate 0.01	G −> A	pos 57	Rate 0.03
C −> G	pos 118	Rate 0.18	G −> C	pos 174	Rate 0.14

Columns 1–3 specify the type of substitution, its position and the substitution rate for the R1 reads. Columns 4–6 detail the respective information for the R2 reads.

Later in this section we will discuss the possibility of motifs causing the accumulation of errors at certain positions as previously reported by Nakamura *et al*. (7) for the Genome Analyzer (GAII).

#### Insertion and deletion error profiles

**Figure 2.**
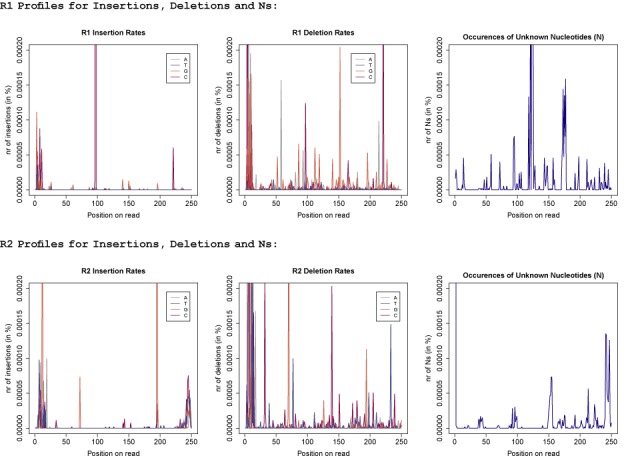
Error profiles for insertions, deletions and unknown nucleotides (Ns): the first three graphs show the R1 error profiles. For insertions the colour identifies the inserted nucleotide and for deletions the colour refers to the type of nucleotide that was deleted. The lower three graphs display the error profiles for the R2 reads, respectively.

Figure 2 displays the position-specific insertion and deletion profiles as well as the distribution of unknown nucleotides (Ns) across all reads. As previously reported the insertion and deletions (indel) rates are ≈100× lower than the substitution rates. We also observed that insertions with rates of 0.000040 and 0.000043 for R1 and R2 reads, respectively, are twice as likely as deletions for which we observed rates of 0.000017 and 0.000027 for R1 and R2 reads, respectively. Again, the majority of indels seem to concentrate around certain positions with rates up to 225× higher than the average indel rate (see Table 2). The non-uniform distributions of unknown nucleotides (N) indicate that Ns as well do not occur randomly.

**Table 2.** Examples of indels occurring at rates considerably higher than the average insertion and deletion rates

Insertions			Deletions		
R1	pos 97	Rate 0.009	R1	pos 221	Rate 0.0003
R2	pos 72	Rate 0.00007	R2	pos 32	Rate 0.0002
R2	pos 195	Rate 0.008	R2	pos 70	Rate 0.0004

#### Correlation of quality scores and errors

**Figure 3.**
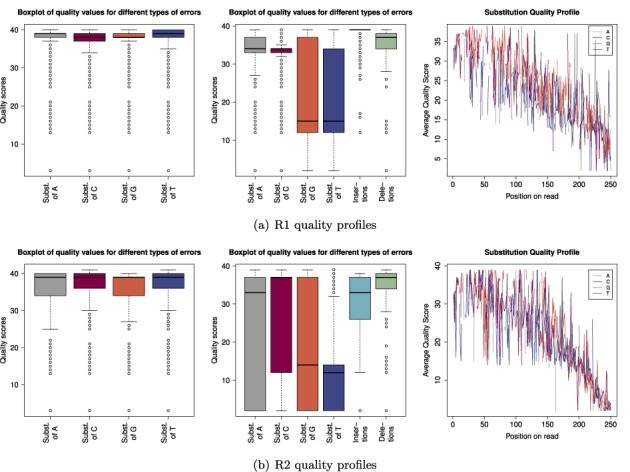
Quality profiles for R1 and R2 reads: the boxplots in the first column display the distribution of quality scores for all reads. The second column shows the distribution of quality scores associated with errors and the last column shows the average quality score of substitution errors for each position across the read.

The first column of Figure 3 displays the observed quality scores for all reads. For this data set we generally encountered very high quality scores for the R1 reads and only slightly lower values for the R2 reads. In the second column of Figure 3 we constrained the boxplot to quality scores associated with substitution errors. Most noticeable is the range of quality scores for substitutions of As and Cs. The average quality score for those types of errors was only slightly lower than the average quality score observed for the respective base in general. Furthermore almost all of the quality scores associated with substitutions of C are between 32 and 35 and 75% of the quality scores associated with substitutions of A are above 32 for the R1 reads (see Figure 3a). The R2 reads showed a larger range for those error types, though a significant number of errors were also associated with very high quality scores. Erroneous Gs and Ts show on average much lower quality values (see Figure 3b). G and T are read by the same laser (green channel). Erroneous bases sequenced on the red channel have on average very high quality values and cannot be detected based on the reported quality score. We observed the same issue for insertions and deletions. In R1 reads 75% of the indels showed quality scores of 35 and above. In R2 reads the same was true for deletions, whereas for insertions the average quality score dropped just below 35. The last column of Figure 3 shows the position-specific substitution quality profiles and suggests that there is a correlation between position of the error and its quality value. Errors occurring at the start and middle of the read had in general much higher quality scores and the quality value decreased towards the end of the reads.

**Figure 4.**
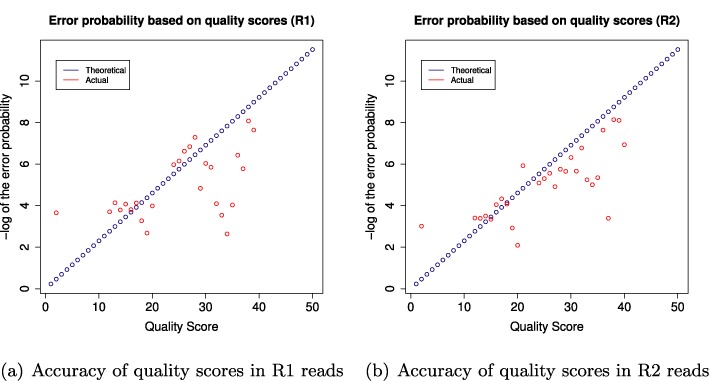
The figure compares the theoretical accuracy (blue) of the quality scores to the actual accuracy (red) observed for data set *DS 35*.

In order to evaluate the suitability of quality scores to identify errors, we compared the theoretical accuracy to the actual accuracy in Figure 4. A quality score *q* is generated for each sequenced base and is designed to predict the probability *p* of an error in the base calling where *q* = −10 · *log*_10_(*p*). The theoretical accuracy *a* is then defined as *a* = 1 − *p*. The actual accuracy is computed by dividing the number erroneous bases which were observed in connection with a certain quality score by the number of times the quality score was observed overall. All displayed quality values of the actual accuracy were observed at least 2000 times. We can see that the theoretical accuracy was higher than the actual accuracy for many of the high quality scores (in particular for the R1 reads), whereas the actual accuracy of the lower quality scores was much higher than the theoretical accuracy.

### Overall comparison of error and quality profiles

We tested a range of factors across 73 data sets including five different library preparation methods, amount of input DNA, number of PCR cycles, two different Taqs, sample/template impact, region (V3 versus V3/V4 and 16S), machine (two different MiSeqs were used), different forward and reverse primers as well as run specificity of the errors. We analysed each data set as described above and computed the corresponding error and quality distributions. We then compared those distributions using the Hellinger distance in order to identify patterns and to determine the experimental factors associated with those patterns. As the Hellinger distance places less emphasis on spikes, there was no need to smooth the distributions prior to computing the distance matrices.

#### Error profiles

**Figure 5.**
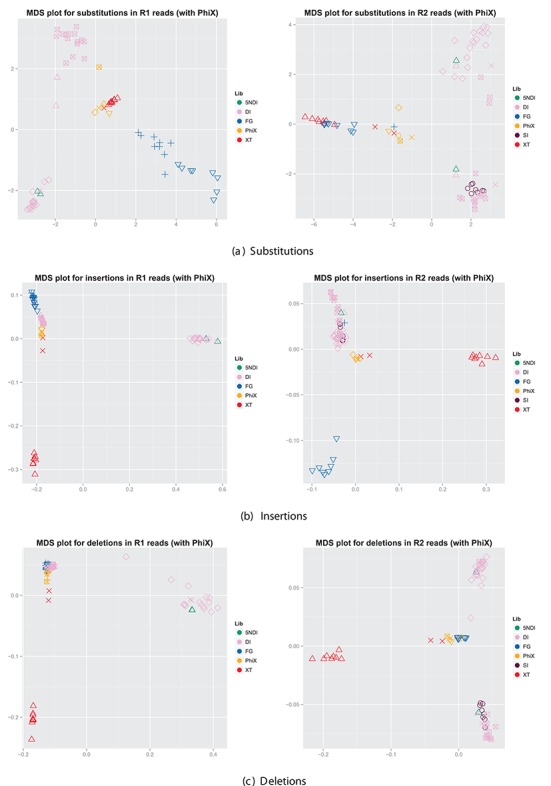
Comparison of error distributions for all data sets. We used the Hellinger distance to construct similarity matrices for the error distributions and summed over all types of substitutions, insertions and deletions, respectively. The colour indicates the library preparation method (see the legend) and the shape indicates different runs.

We visualized the level of similarity of the position-specific error distributions by means of multidimensional scaling (MDS) (see Figure 5). A distance matrix was computed based on the pairwise comparison of the error distributions for each data set using the Hellinger distance. MDS is a technique that visualizes the information in the distance matrix by computing the relative position of the objects in a two-dimensional space whilst aiming to preserve the original distances. Therefore the closer objects are in the MDS plot, the more similar they are. In order to derive meaningful error distributions for a data set, we required at least 1000 aligned reads per data set. (Note that none of the SI data sets held ≥1000 aligned R1 reads. The SI data sets were thus not included in the R1 figures.) Across all types of errors there was a distinct tendency to cluster according to library preparation (indicated by colour) and run (indicated by shape). The R1 substitution profiles for the FG data sets, for example, formed a distinct cluster. This cluster consists, in turn, of two subclusters reflecting that the samples were sequencing on two different runs. The DI and 5NDI data sets clustered as well though we observed a higher degree of variability between different sequencing runs. The XT data sets clustered tightly aside from two data points representing the full-length 16S samples. The PhiX data sets from each run formed their own distinct cluster.

For both R1 and R2 substitutions the permutation ANOVA identified the library preparation method as the major factor, explaining 37% of the variability and 44%, respectively. (For further details see Supplementary Tables S7 and S8.)

#### Quality profiles

We repeated the analysis for the quality profiles and the corresponding MDS plots can be found in the Supplementary material (see Supplementary Figure S3). We observed a similar tendency for the substitution profiles to cluster with regard to the library preparation method. The quality scores for the insertions and deletions showed less of a pattern.

### Comparison of error rates for different library preparation methods

**Figure 6.**
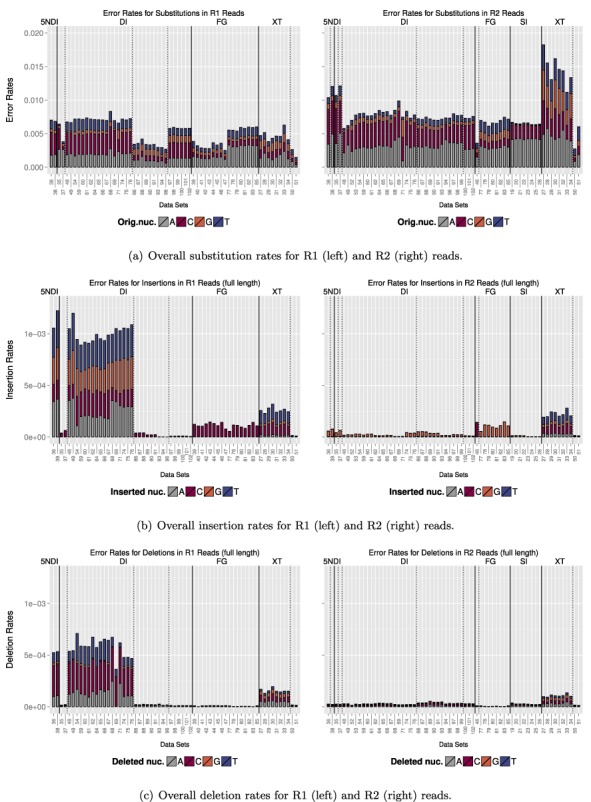
We compared the overall error rates for each data set. The lower x-axis indicates the name of the data set and the upper x-axis specifies the library preparation method. The error bars show the extent that each original nucleotide contributed to the overall error rate. Data sets are grouped by library preparation (solid lines) and primers (dashed lines).

In Figure 6 we compared the overall error rates of the data sets grouped by library preparation method. Note that we only considered aligned reads here. An overview of the percentage of aligned reads is given in Figure 11 (rates for raw reads are marked in grey). For all of the data sets the error rates increased for the R2 reads. We noted the most dramatic increase for some of the XT data sets where the error rate for the R2 reads was more than double the rate of the R1 reads. We also noticed a certain amount of variation for each library preparation method. In the case of the FG data sets, for example, *DS39*-*DS47* were on the same sequencing run and showed a lower rate compared to the other FG data sets that were sequenced on a different run. For the DI data sets four different forward primers were used. The first two data sets, the following 17 data sets, the following nine data sets and the last six data sets have the same forward primer, respectively. There was also a clear bias of A and C which, in particular for the R2 reads, accounted for a large fraction of the overall error rate. Note that A and C are both read by the same laser (red channel).

**Figure 7.**
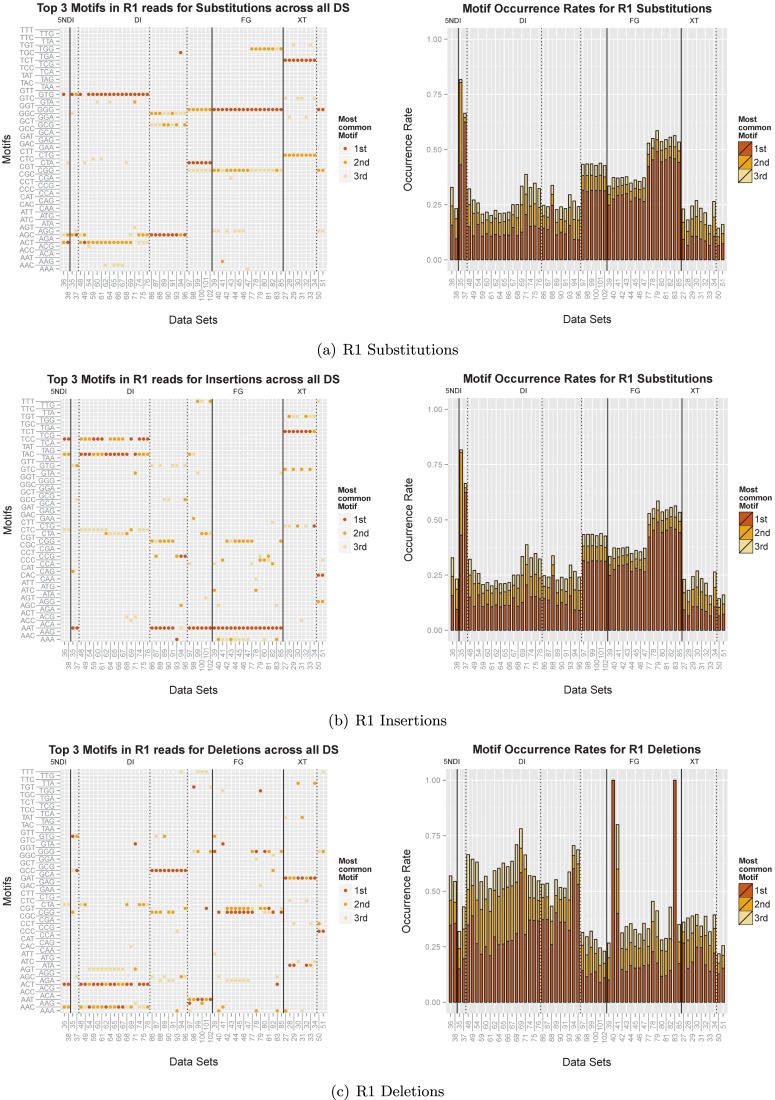
We recorded all 3 mers preceding substitution, insertion or deletion errors, respectively. The first column displays the three most common motifs for each data set and the second column illustrates the percentage of errors that were associated with the respective motif. The solid lines separate the data sets according to library preparation methods and the dashed lines further divide them according to the different forward primers that were used.

**Figure 8.**
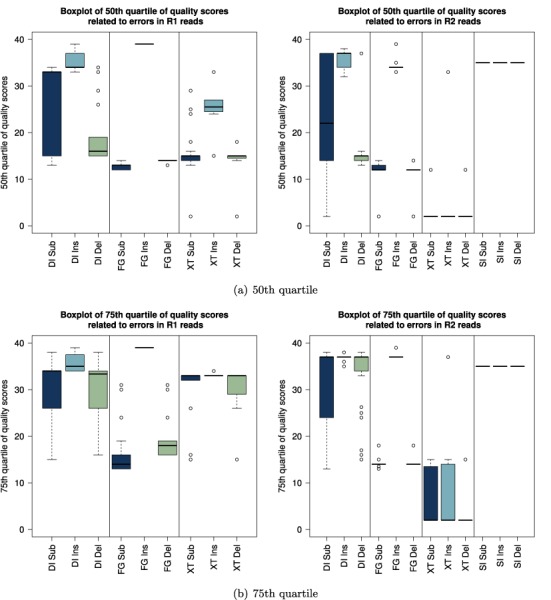
Overview of 50th and 75th quartile of quality scores associated with errors across all data sets. The results for the R1 reads are displayed on the left and the results for the R2 reads are on the right. Data sets were grouped by library preparation method (DI = dual index; SI = single index; FG = Fusion Golay; XT = NexteraXT) and substitution, insertion and deletion errors are displayed separately. Note, that for none of the single index data sets enough R1 reads aligned to construct meaningful quality profiles (threshold = 1000 reads).

**Figure 9.**
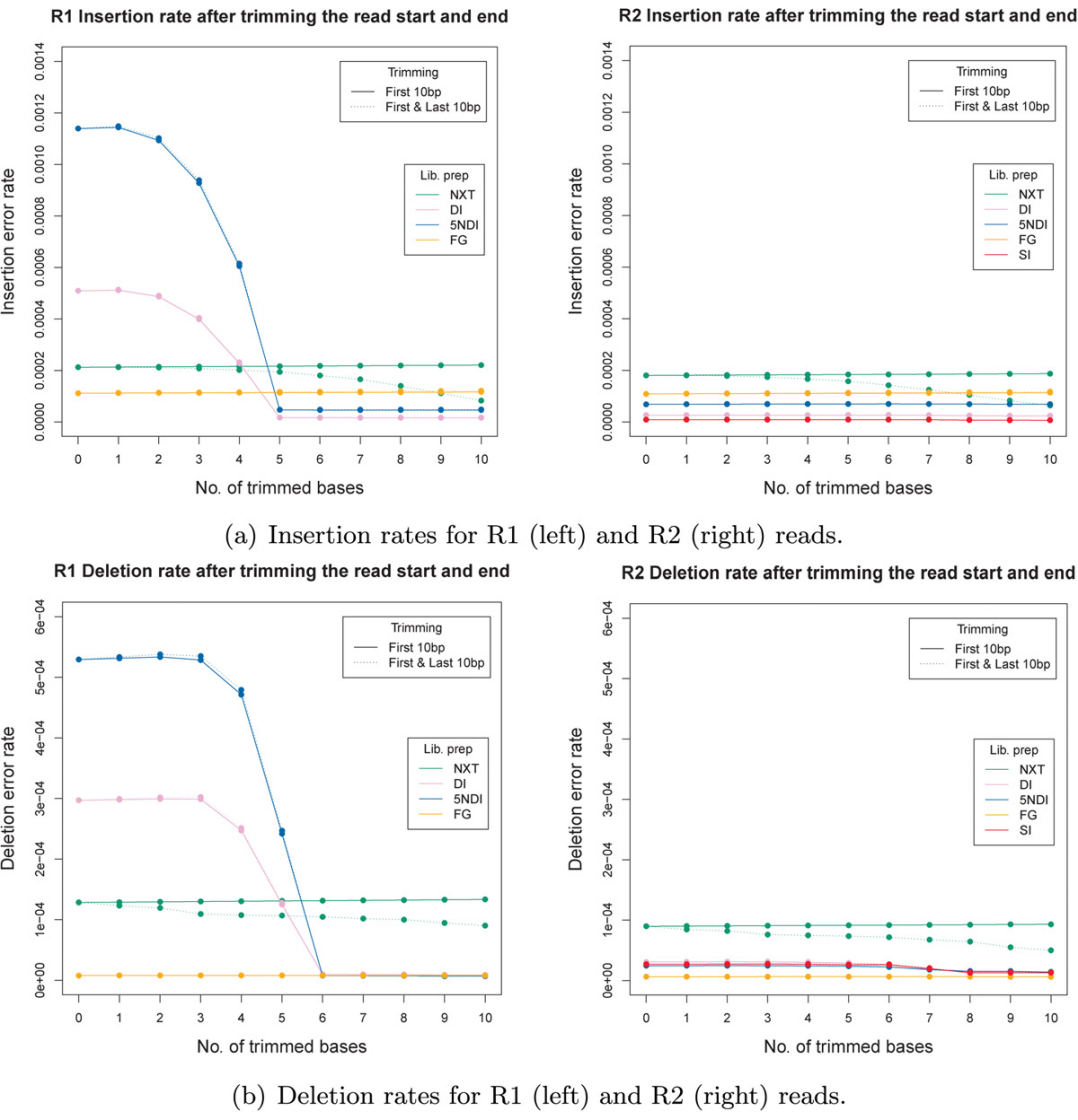
Insertion and deletion rates of raw reads, after trimming up to 10 bp off the read start and after additionally trimming up to 10 bp off the read end. (Note: none of the R1 single index data sets contained ≥1000 reads after alignment.)

**Figure 10.**
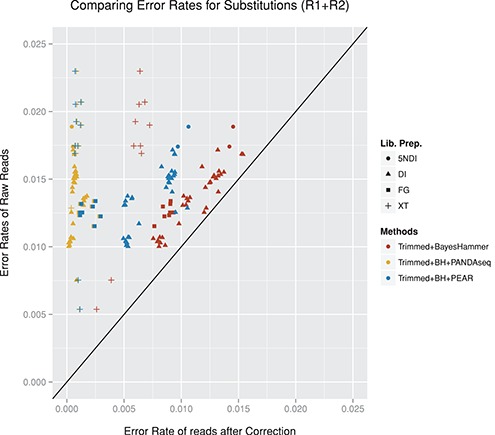
The figure compares the error rates of the raw reads (R1+R2 rates) to different error corrections approaches including Trimming+BayesHammer, overlapping reads with PANDAseq and overlapping reads with PEAR. We only included data sets for which at least 1000 reads aligned for all methods. Data sets not included: 19–26, 52+53 (not enough raw R1 reads aligned), 39–45+47 (not enough raw R2 reads aligned).

**Figure 11.**
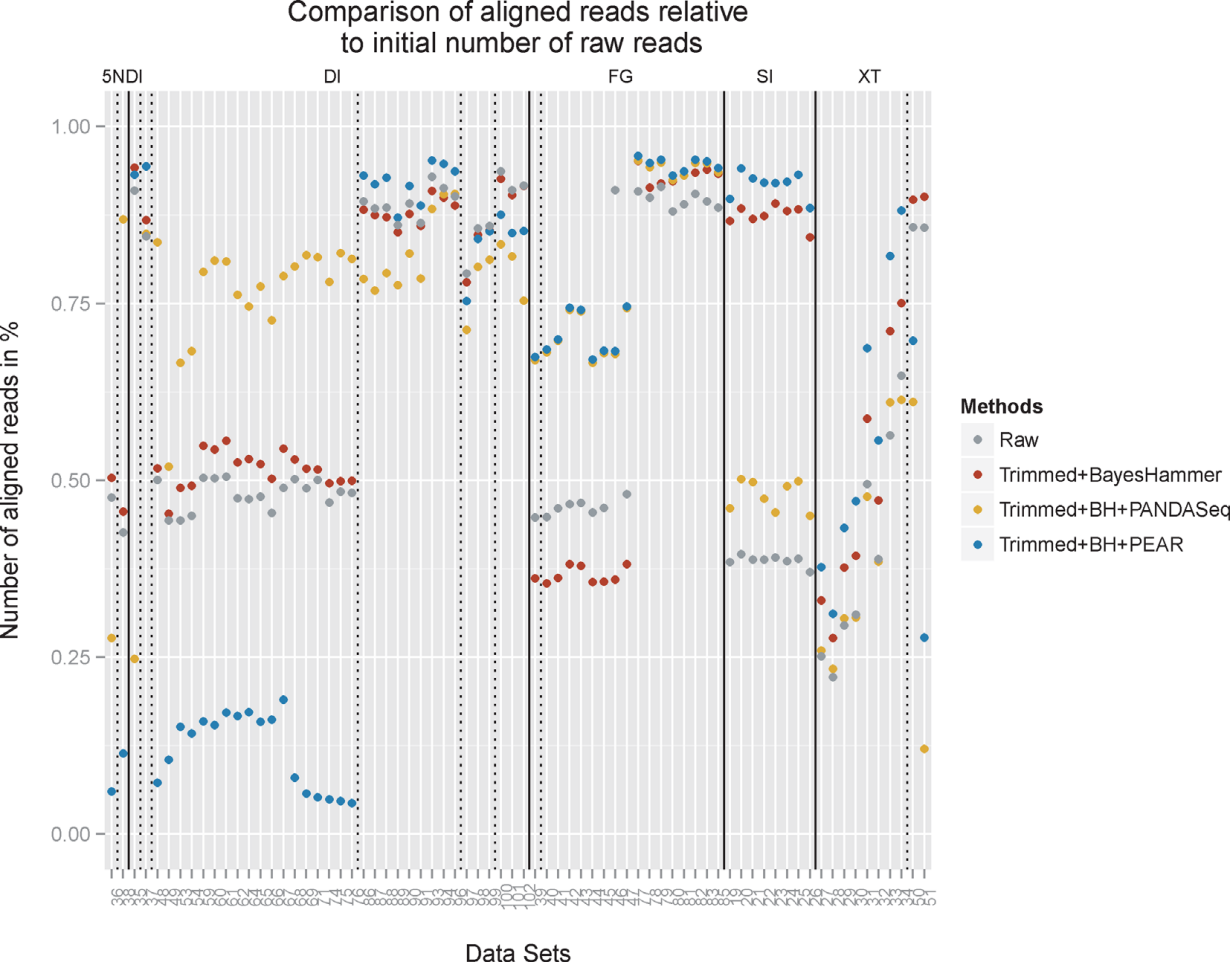
The figure compares the number of aligned reads relative to the initial number of raw reads. For the raw reads and for reads that were processed with Sickle plus BayesHammer, we summed the R1 and R2 rates. We also included trimming plus BayesHammer and overlapping with PANDAseq and PEAR, respectively, as those combination of approaches returned the lowest error rates. Data sets are grouped by library preparation (solid line) and primers (dashed line).

Indel rates are in general almost two orders of magnitude smaller than the substitution rates. Though for 17 of the DI data sets and the two 5NDI data sets we recorded a huge increase in insertions and deletions on the R1 reads. For all of these data sets the 515 or F515A forward primer was used. For the data sets where the same forward primer was used in connection with the FG we did not record the same build-up of indels. The majority of those errors occurred at the start of the reads. By trimming the first 10 bp of the reads we were able to remove 95–100% of all insertion errors for those data sets and 96–100% of all deletion errors (see Supplementary Figure S4).

We also detected a preference for the substituting nucleotide for the different library preparation methods. (For more details see Supplementary Figures S5 and S6.) This bias seems to be mostly run specific, though we recorded a high preference for G as the substituting nucleotide in R1 reads and T in R2 reads for the Fusion Golay. For *DS82*, for example, G was the substituting nucleotide in 68% of all substitutions that occurred on R1 reads and T in 65% of all substitutions that occurred on R2 reads.

### Motifs

We recorded all 3mers preceding errors (in the following referred to as ‘motifs’) for substitutions, insertions and deletions and measured the percentage of errors that is explained by the top three motifs. Figure 7 shows the results for the R1 reads. (The analogous results for the R2 reads can be found in Supplementary Figure S7.)

In particular for the substitutions, Figure 7a and Supplementary Figure S7a show that the three most common motifs are very similar for data sets with the same library preparation (separated by the solid lines) with additional subclusters based on the forward and reverse primers (indicated by the dashed lines), respectively. The plots on the right side show in each case the percentage of substitution errors that follow motif1, motif2 and motif3, respectively. In the case of *DS35* more than 80% of all errors succeed ‘GTG’ (motif1) or ‘AGC’ (motif2). And for half of the FG data sets only three motifs (out of 64 possible ones) account for more than 50% of all substitution errors. On average the three most common motifs accounted for 34% of all substitution errors. This bias is even more pronounced for insertions, where more than 95% of all errors are preceded by the motif ‘AAT’ for all FG data sets. And on average 72% of all insertion errors follow the three most common motifs. For deletions we were able to connect on average 48% of all errors to three motifs. For the R2 reads the motifs account for an even larger fraction of errors in the case of substitutions and insertions: on average 44% of all substitution errors, 78% of all insertion errors and 46% of all deletion errors can be connected to three motifs. And more than 95% of all insertion errors in the FG data sets were related to a single motif.

To determine the driving factors for the formation of these motifs, we used a permutation ANOVA with the Bray–Curtis distance analogously to the analysis of the error profiles. For the R1 and R2 substitutions the forward and reverse primer combination explains the largest fraction of the variance with 60 and 55%, respectively. The library preparation method explains an additional 16 and 26%, respectively. For insertions a total variance of 78 and 80%, respectively for R1 and R2 reads, can be explained by primers together with the library design and 78% for R1 deletions. The significant factors for R2 deletions are the run and the library design explaining a total of 25% of the variance.

#### Quality scores

We investigated the quality scores associated with errors across all data sets. Figure 8a and b display the 50th and the 75th quartile for all data sets, meaning 50 and 25%, respectively, of all quality scores associated with errors were above these values. The data sets are grouped by library preparation and the quality scores associated with substitutions, insertions and deletions are displayed separately. For the DI data sets a large fraction of errors showed high quality scores. In particular, 50% of all R1 and R2 insertions were connected with quality scores of 32 and above for all data sets. For the FG data sets substitutions and deletions were well characterized by their quality scores but insertions showed very high quality scores. For the XT data sets we recorded high quality scores for ≥25% of all errors. However, the 50th quartile was overall lower than for the DI data sets and errors on R2 reads were well characterized. The SI data sets showed very high quality scores across all types of errors.

### Error correction

We compared different error removal techniques including trimming the start and end of the reads, trimming based on quality scores with Sickle (https://github.com/najoshi/sickle), error correction with BayesHammer (13) and overlapping reads with PEAR (v0.9.1) (14) or PandaSeq (Version 2.4, with a minimum overlap of 50 bp for V4 data sets and 10 bp for V3/V4 data sets) (15) and combinations of the different strategies.

#### Insertions and deletions

Trimming the start and/or end of the reads proved to be an important step in removing indel errors for data sets prepared with certain library preparation methods. The average error rates for the different library preparation methods are shown in Figure 9. Here, we compare the indel error rates of the raw reads to the rates after trimming up to 10 bp off the read start and after additionally trimming up to 10 bp off the read end. For XT on average 61% of the insertion and 29% of the deletions can be removed from the R1 reads by trimming the last 10 bp and 64 and 44%, respectively, for the R2 reads. Trimming the start of the read had no significant effect on the indel error rates for this library preparation method (see the solid green line in Figure 9). For the DI data sets trimming the start of the read removed most indel errors. On average 97% of the R1 insertion in DI data sets and 96% in the 5NDI data sets could be removed by trimming the first 5 bp of the read. Additional trimming at the end showed no further improvements. R1 deletion rates could on average be reduced by 97 and 99% for the DI and 5NDI data sets, respectively, by trimming the first 6 bp of the reads.

#### Substitutions

By overlapping the reads we were able to achieve further significant improvements with regard to the error rates. The best results in terms of error removal were achieved with a combination of quality trimming the reads with Sickle, then applying BayesHammer for error correction and then overlapping the reads with PANDAseq (see Supplementary Figure S11 for details on PANDAseq). For the data sets displayed in Figure 10 the substitution error rates were reduced by 77–98% with an average of 93.2%. Figure 11 compares the percentage of aligned reads for the most successful approaches. PANDAseq was able to align between 12 and 95% of the reads with an average of 69% across all data sets.

PEAR was able to reduce error rates by about 60% on average (range 18–97%). The number of aligned reads ranged from 4 to 96% and on average 61% of the read-pairs could be aligned after overlapping. PEAR encountered problems with the alignment of the DI and 5NDI data sets with high indel rates. For the FG data sets PANDAseq and PEAR produced similar results in terms of aligned reads with lower rates for *DS 39-47* where the fraction of substitutions linked to the top three motifs was about 20% lower. It is also noticeable that for these data sets quality trimming combined with error correction lowered the number of aligned reads by ∼15% on average. PANDAseq also encountered problems with the SI data set with lower rates than trimming+BayesHammer and trimming+BayesHammer+PEAR. The XT data sets produced very mixed results with regard to the percentage of aligned reads. The best results for the XT amplicon data sets were achieved by PEAR which aligned between 31 and 88%. Note that *DS 50* and *51* were the full-length 16S data sets (displayed in the last two columns of Figure 11). For *DS 50* fragments between 500 and 1000 bp were selected (average 590 bp) and for *DS 51* fragment size selection included sequences between 600 and 1500 bp (average 767 bp). Although smaller fragments will preferentially be sequenced, we would expect (in particular for *DS 51*) that only a small fraction of the reads can be overlapped.

If read-overlapping is not a possibility (i.e. if the average fragment size was larger than two times the read length), the best strategy for error removal was quality trimming followed by error correction with BayesHammer (see Supplementary Figures S8 and S9). We recorded the most substantial improvement for the R2 reads of the XT data sets. The error rates slightly increased for some of the data sets after quality trimming. This is due to an increase in the number of aligned reads. When restricting the data sets to the reads that aligned prior to trimming/correcting the reads, the rates very slightly decreased.

## DISCUSSION

We have shown that the experimental design has a major impact on the error patterns of the sequencing data. To our knowledge, this was the first study on error profiles for the MiSeq and also the first time that a large range of experimental factors was tested in connection with error patterns. We used a complex mock community, to reflect the conditions encountered in real samples, as well as single species. A total of 73 data sets were used to show that the library preparation method together with the choice of primers causes an extensive bias towards certain motifs causing substitutions, insertions and deletions, respectively. This provides strong evidence that Illumina errors do not occur randomly.

The increased error rates that we observed towards the end of the reads are assumed to be due to accumulation of phasing and pre-phasing events throughout the sequencing process. Every time a molecule fails to elongate properly or advances too fast, the overall signal for the cluster suffers from interference. So as the read length increases, the cluster signal can get weaker due to an accumulation of these events resulting in higher error rates towards the end of the read (16). This explains the gradual increase of errors that we observed in the position and nucleotide-specific distributions in addition to the spikes caused by the motifs.

We demonstrated that A and C are more prone to substitution errors compared to G and T. Both A and C are identified through the red channel. This indicates a problem with either the red laser or the filter that is used to distinguish between the two nucleotides. Also, the fluorescence emitted by A and C have the highest intensities. So any interference with the signal would result in an erroneous base call. G on the other side shows the lowest initial emission intensity. In particular for the Fusion Golay, the most common substituting nucleotide was a G which could also indicate signal disturbances.

Another possible explanation for the spikes that were observed in the individual position and nucleotide-specific error profiles are PCR errors. Again, we believe that this is unlikely to be the cause for the majority of the spikes as the error rates representing the spikes (see Table 1) exceed the abundance level of the individual 16S sequences in the mock community. PCR errors would need to occur in multiple 16S sequences at the same position.

Our quality score analysis showed that quality scores are of limited use for the identification of errors in amplicon sequencing data. Results differed for the various library preparation methods. Only substitutions and deletions in the FG data sets and errors in the R2 reads for the XT library preparation method were well characterized, whilst the majority of errors for all other library preparation methods was associated with high quality scores. Kozich *et al*. (17) had previously reported that errors were rarely associated with quality scores above 21. However, a significant fraction of the errors is expected to arise during the PCR amplification step and would therefore not necessarily be associated with low quality scores. We were not able to confirm their results in our study. This is in accordance with the results by Eren *et al*. (18) who studied *Escherichia coli* V6 amplicons in connection with the use of fusion primers.

We have shown that the theoretical accuracy indicated by the quality scores was higher than the actual accuracy observed for data set *DS 35*. It is possible that some of the high quality scores associated with errors refer to PCR errors introduced before the actual sequencing step. Nevertheless these results indicate that quality scores are of limited use for the identification of errors in this amplicon data set as low quality values do not reliably reflect the error potential of the respective base.

Since the study presented by Nakamura *et al*. (7) on the Illumina Genome Analyzer (GAII) in 2011 there have been major developments with another four Illumina platforms entering the market and improvements regarding the chemistry providing much longer reads with lower error rates. Nakamura's findings are based on a single library preparation method and the read length was limited to 36 bp. We tested if a similar bias prevails for the MiSeq platform with read lengths of 2 × 250 bp testing five different library preparation methods. We additionally assessed the impact of different environmental factors. Besides the library preparation method, we identified the forward and reverse primers as one of the major driving factors for the error profiles. The sources of errors described above (i.e. phasing and pre-phasing, problems with red laser/filter) can be attributed to the actual sequencing process. In contrast to this, the library preparation method and the choice of primers are biases introduced prior to the sequencing process.

Figure 5 showed that the error profiles of the PhiX data sets formed their own distinct cluster and therefore significantly differ from the other data sets. This is in accordance with the assumption that the library preparation has a major impact on the error distribution as the adapters used for PhiX are the same as for the TruSeq library preparation method and would thus show a distinct pattern. This also implies that PhiX is not suitable to identify error rates or patterns if the actual sample was prepared with a different library preparation method.

**Figure 12.**
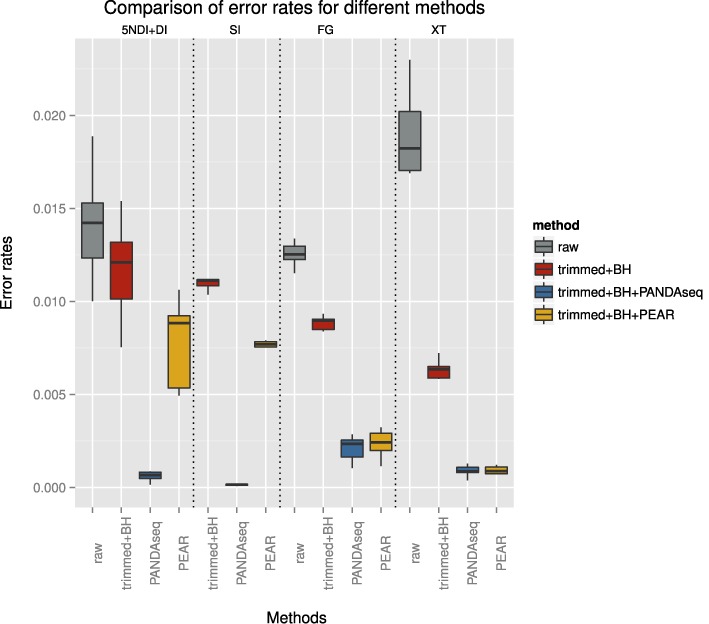
The figure shows the range of average error rates for the different library preparation methods (indicated on the upper x-axis). The grey bar plots show the error rates for the raw reads, in red are the error rates after trimming and error correction, in blue and yellow are the error rates after additionally overlapping reads with PANDAseq and PEAR, respectively.

Figure 12 summarizes the error rates for each library preparation method with regard to different error removal techniques. For none of the SI data sets could we align enough of the raw R1 reads. Overall the highest error rates were encountered for the XT data sets. However, trimming plus error correction achieved very good results on these data sets as well as additionally overlapping reads resulting in error rates comparable to the other library preparation methods. PANDAseq achieved the best results across all library preparation methods. Overall the figure shows that error rates can be significantly reduced by combining various strategies for error removal.

The large fraction of errors that are not sufficiently characterized by quality scores pose the main limitation for approaches such as quality trimming and error correction. This has been previously reported for the V6 region of *E. coli* amplicon sequencing data (18). Much better rates were achieved through read overlapping as these approaches not only rely on the quality score but also utilize information from both read directions. Also, this approach particularly addresses errors at the end of the reads where error rates are significantly higher compared to the start of the read. Motif-based errors are not addressed directly. However, as different motifs were recorded for forward and reverse reads, any systematic error is likely to be observed only on one read direction and can therefore be addressed indirectly.

Choosing the most appropriate approach for a particular data sets depends on the individual hypothesis. We need to balance error rate reduction with maximization of aligned reads. For this we need to take the experimental factors into account. The number of aligned reads increased after trimming plus error correction for most data sets. Error rates were slightly reduced for the R1 reads and significantly reduced for the R2 reads. Thus quality trimming and error correcting reads is sensible for any kind of data sets. Indel errors for the DI (DI and 5NDI) data sets accumulated over the first 6 bp of the reads. Trimming of the start of the reads removed on average 97% of all indel errors for these data sets. Overlapping reads with PANDAseq has proven most effective in removing errors but might reduce the number of aligned reads depending on the library preparation method and primers that were used. PEAR achieved higher numbers of aligned reads for some data sets but was not able to reduce errors to the same extent.

We observed similar results in terms of errors, motifs, read alignment and error removal potential for data sets with similar experimental design, i.e. the same library preparation method, forward and reverse primers and sequenced on the same run. Including a small mock community in a sequencing run could thus be used to determine the best strategy for removing errors from the sequencing data. We showed that PhiX is not suitable for this as the adapters used for PhiX represent a specific library preparation method that can differ from the one used for the actual sample. The purpose of PhiX is often to increase the data quality of low diversity samples and to optimize the cluster map generation (http://res.illumina.com/documents/products/technotes/technote_phixcontrolv3.pdf). The same can be achieved by including a mock community with the added benefit of detailed information on the error patterns.

Sequencing errors caused by motifs are more noticeable in amplicon data sets because of a higher degree of similarity between the sequences. They are represented by spikes in the position-specific error distributions. We will subsequently extend our study to metagenomic data sets. This will allow us to separate sequencing errors from PCR errors and give further insight into the sources for different types of miscalls.

Systematic errors can cause major problems during the analysis of the sequencing data if programs assume that errors occur randomly. In particular for the identification of single nucleotide polymorphisms (SNPs) systematic errors will result in a high false positive rate and for diversity estimates systematic errors might result in a significant overestimation of the diversity in the sample. In order to identify these systematic errors, it is important to infer individual error profiles for different sequencers, library preparation methods and sequencing types to handle miscalls. Illumina error rates are currently based on errors detected for the PhiX genome during the sequencing process. We showed that these error rates can greatly differ from the actual sample. Our approach offers the possibility to infer detailed error profiles for individual sequencing runs.

## AVAILABILITY

The code is available on: https://bitbucket.org/ms_research/ep.

## ACCESSION NUMBER

The data are available on the European Nucleotide Archive under the study accession number: PRJEB6244 (http://www.ebi.ac.uk/ena/data/view/PRJEB6244).

## SUPPLEMENTARY DATA

Supplementary data are available at NAR Online.

SUPPLEMENTARY DATA
