# Supplementary material for: Insight into biases and sequencing errors for amplicon sequencing with the Illumina MiSeq platform
Source: Nucleic Acids Res. 2015 Jan 13;43(6):e37. doi: 10.1093/nar/gku1341 (PMC4381044; doi:10.1093/nar/gku1341)
Supplement: SUPPLEMENTARY DATA [file supp_43_6_e37__index.html]

Insight into biases and sequencing errors for amplicon sequencing with the Illumina MiSeq platform — SUPPLEMENTARY DATA 

# Insight into biases and sequencing errors for amplicon sequencing with the Illumina MiSeq platform

## SUPPLEMENTARY DATA

**Files in this Data Supplement:**

- SUPPLEMENTARY DATA
