# Supplementary material for: Insight into biases and sequencing errors for amplicon sequencing with the Illumina MiSeq platform
Source: Nucleic Acids Res. 2015 Jan 13;43(6):e37. doi: 10.1093/nar/gku1341 (PMC4381044; doi:10.1093/nar/gku1341)
Supplement: SUPPLEMENTARY DATA [file supp_gku1341_nar-01235-met-n-2014-File003.pdf]

Supplementary Material

Comparing Different Library Preparation Methods

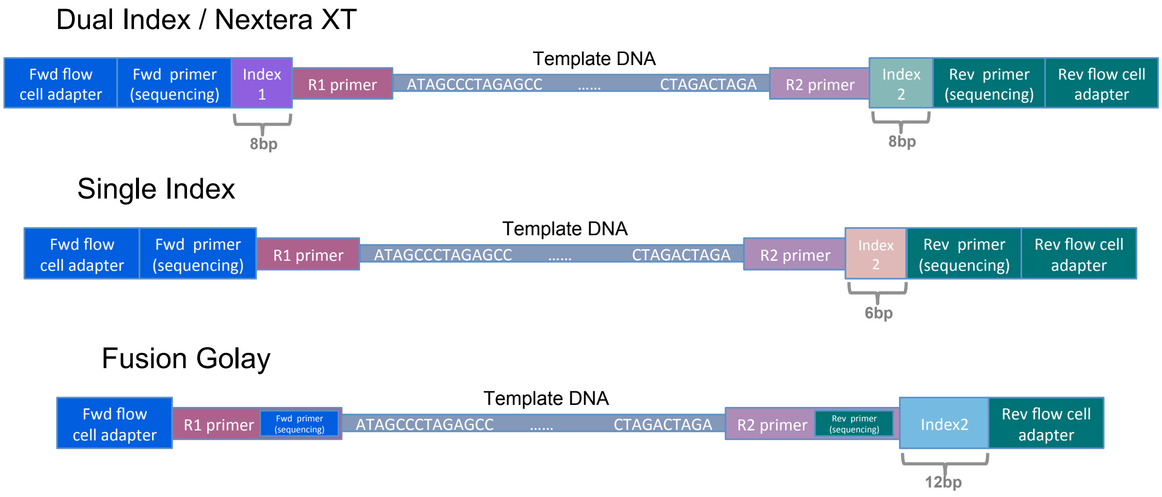

Figure S1: Overview of the different amplicon design methods.

Fusion Golay Primer Design:

1-10 ng of balanced or unbalanced synthetic community is subjected to an amplification step using 0.3 μM primer forward fusion; 0.3 μM primer reverse fusion specific; 1x HiFi or Q5 polymerase ready mix. The PCR for each variable region was carried out in triplicate in a 25 μl reaction in a Thermal Cycler (Applied Byosistem GeneAmp PCR system 9700) with the following parameters: initial denaturation at 94°C for 5 min, followed by 25 cycles of 98°C for 20 s, 60°C for 15 s, and 72°C for 40 s with a final extension at 72°C for 1 min.

Nextera:

1 ng of balanced or unbalanced synthetic community DNA is submitted to the following amplification reaction: 0.3 μM forward primer (27YMF); 0.3 μM reverse primer (1492R); 1x HiFi polymerase ready mix. The PCR was carried out in triplicate in a 25 μl reaction in a Thermal Cycler (Applied Byosistem GeneAmp PCR system 9700) with the following parameters: initial denaturation at 94°C for 5 min, followed by 25 cycles of 98°C for 20 s, 60°C for 15 s, and 72°C for 40 s with a final extension at 72°C for 1 min. The amplicon libraries were cleaned to remove excess nucleotides, salts and enzymes using 20 μl of the Agencourt AMPure XP system (Beckman Coulter Genomics) and eluted in TE buffer. 50 ng of amplicon library was submitted to a Nextera DNA library preparation following the Nextera ® DNA Sample Preparation Guide recommendation.

Universal Tailed Tag design: (Single or dual index barcoding strategy)
1-10 ng of balanced or unbalanced synthetic community DNA was used in the first amplification step using the following reaction: 0.1 μM forward primer (F515A, 515, 341f); 0.1 μM reverse tailed primer (806rcb, 805R); 1x HiFi or Q5 polymerase ready mix. The PCR was carried out in triplicate in a 25 μl reaction in a Thermal Cycler (Applied Byosistem GeneAmp PCR system 9700) with the following parameters: initial denaturation at 94°C for 5 min, followed by 10 cycles of 98°C for 20 s, 60°C for 15 s, and 72°C for 40 s with a final extension at 72°C for 1 min. The amplicon libraries were cleaned to remove excess nucleotides, salts and enzymes using 20 μl of the Agencourt AMPure XP system (Beckman Coulter Genomics) and eluted in 10 μl of TE buffer. The 10 μl of the first step reaction was submitted to a second amplification step using the following condition: 0.1 μM forward barcoded primer (DI_MIDFor) for the dual index strategy or a forward not barcoded primer for the single index strategy; 0.1 μM primer barcoded reverse primer (DI_MIDRev); using same cycling condition as above.

Table S1: PCR primers used in this study: The variable region primer sequence is underlined and in bold. The position of the multiplex identifier (MID) is shown as [x] . Standard and Nextera MID sequences were used for single and dual index libraries while for Fusion Golay a 12 bp corrected MID was used as shown in Table S2. Degenerated bases in the sequence represent the following nucleotides: M: C or A; B: not A; Y: C or T; R: A or G; W: A or T; H: not G; K: G or T; V: not T.

Primer name	Library design	Variabel region	Sequence
27YMF<br>1492R	NEXTERA	V1-V9	AGAGTTTGATYMTGGCTCAG<br>TACGGYTACCTTGTTAYGACTT
F515A<br>805R	DI	V4	CTACACTCTTTCCCTACACGACGCTCTTCCGATCT**GTGBCAGCMGCCGCGGTAA**<br>GTGACTGGAGTTCAGACGTGTGCTCTTCCGATCT**GACTACHVGGGTATCTAATCC**
DI_MIDFor<br>DI_MIDRev	DI	V4	AATGATACGGCGACCACCGAGATCTACACxxxxxxxx**ACACTCTTTCCCTACACGACG**<br>CAAGCAGAAGACGGCATACGAGATxxxxxxxx**GTGACTGGAGTTCAGACGTGTGCTCTTCCGATCT**
314F<br>806rbcx	DI	V3-V4	CTACACTCTTTCCCTACACGACGCTCTTCCGATCTCCTAYGGGRBGCASCAG<br>CAAGCAGAAGACGGCATACGAGATxxxxxxxxxxxx**AGTCAGTCAGCCGGACTACHVGGGTWTAA**
515<br>806rcbcx	FG	V4	AATGATACGGCGACCACCGAGATCTACACTATGGTAATTGT**GTGCCAGCMGCCGCGGTAA**<br>CAAGCAGAAGACGGCATACGAGATxxxxxxxxxxxx**AGTCAGTCAGCCGGACTACHVGGGTWTAAT**

Table S2: An error corrected multiplex identifier sequence used with MiSeq sequencing technology. A 12bp reverse Index used for unidirectional tagging in the Fusion Primer approach (F).

Primer name	Library design	Sequence
806rcbc0	FG	TCCCTTGTCTCC
806rcbc1	FG	ACGAGACTGATT
806rcbc2	FG	GCTGTACGGATT
806rcbc3	FG	ATCACCAGGTGT
806rcbc4	FG	TGGTCAACGATA
806rcbc5	FG	ATCGCACAGTAA
806rcbc6	FG	GTCGTGTAGCCT
806rcbc7	FG	AGCGGAGGTTAG
806rcbc8	FG	ATCCTTTGGTTC
806rcbc9	FG	TACAGCGCATAC
806rcbc10	FG	ACCGGTATGTAC
806rcbc11	FG	AATTGTGTCGGA
806rcbc12	FG	TGCATACACTGG
806rcbc13	FG	AGTCGAACGAGG
806rcbc14	FG	ACCAGTGACTCA
806rcbc15	FG	GAATACCAAGTC
806rcbc16	FG	GTAGATCGTGTA
806rcbc17	FG	TAACGTGTGTGC
806rcbc18	FG	CATTATGGCGTG
806rcbc19	FG	CCAATACGCCTG
806rcbc20	FG	GATCTGCGATCC
806rcbc21	FG	CAGCTCATCAGC
806rcbc22	FG	CAAACAACAGCT
806rcbc23	FG	GCAACACCATCC

Primer name	Library design	Sequence
806rcbc24	FG	GCGATATATCGC
806rcbc25	FG	CGAGCAATCCTA
806rcbc26	FG	AGTCGTGCACAT
806rcbc27	FG	GTATCTGCGCGT
806rcbc28	FG	CGAGGGAAAGTC
806rcbc29	FG	CAAATTCGGGAT
806rcbc30	FG	AGATTGACCAAC
806rcbc31	FG	AGTTACGAGCTA
806rcbc32	FG	GCATATGCACTG
806rcbc33	FG	CAACTCCCGTGA
806rcbc34	FG	TTGCGTTAGCAG
806rcbc35	FG	TACGAGCCCTAA
806rcbc36	FG	CACTACGCTAGA
806rcbc37	FG	TGCAGTCCTCGA
806rcbc38	FG	ACCATAGCTCCG
806rcbc39	FG	TCGACATCTCTT
806rcbc40	FG	GAACACTTTGGA

Overview Data Sets

Table S3: Overview of the experimental design for the data sets. Library preparation methods: nested single index (SI), NexteraXT (XT), nested dual index (DI), nested dual index with 5 random nucleotides before primer (5NDI), Fusion Golay (FG); Taq: HiFI Kapa (HF), Q5 neb (Q5); Template: *Anaerocellum thermophilum Z-1320 DSM 6725* (AT), *Bacteroides thetaiotaomicron VPI-5482* (BT), *Bacteroides vulgatus ATCC 8482* (BV), *Caldicellulosiruptor saccharolyticus DSM 8903* (CS), *Herpetosiphon aurantiacus ATCC 23779* (HA), *Rhodopirellula baltica SH 1* (RBS), *Leptothrix cholodnii SP-6* (LC), balanced mock community (MB) , unbalanced mock community (MUB); Primers: see Table S1+S2 for sequences

Meta ID	Lib. Prep.	Run	Region	Machine	input ng	PCR cycle (R1+R2)	Taq	Template	F & R primer
19	SI	1	V4	Miseq2	4	12+15	Q5	AT	515 & 805RA
20	SI	1	V4	Miseq2	4	12+15	Q5	BT	515 & 805RA
21	SI	1	V4	Miseq2	4	12+15	Q5	BV	515 & 805RA
22	SI	1	V4	Miseq2	4	12+15	Q5	CS	515 & 805RA
23	SI	1	V4	Miseq2	4	12+15	HF	AT	515 & 805RA
24	SI	1	V4	Miseq2	4	12+15	HF	BT	515 & 805RA
25	SI	1	V4	Miseq2	4	12+15	HF	BV	515 & 805RA
26	SI	1	V4	Miseq2	4	12+15	HF	CS	515 & 805RA
27	XT	2	V3/V4	Miseq1	2	15+12	Q5	AT	341f & 806rcb
28	XT	2	V3/V4	Miseq1	2	15+12	Q5	BT	341f & 806rcb
29	XT	2	V3/V4	Miseq1	2	15+12	Q5	BV	341f & 806rcb
30	XT	2	V3/V4	Miseq1	2	15+12	Q5	CS	341f & 806rcb
31	XT	2	V3/V4	Miseq1	2	12+12	HF	AT	341f & 806rcb
32	XT	2	V3/V4	Miseq1	2	12+13	HF	BT	341f & 806rcb
33	XT	2	V3/V4	Miseq1	2	12+14	HF	BV	341f & 806rcb
34	XT	2	V3/V4	Miseq1	2	12+15	HF	CS	341f & 806rcb
35	DI	2	V4	Miseq1	2	12+18	HF	MB	515 & 805RA
36	5NDI	2	V4	Miseq1	2	12+18	HF	MB	515 & 805RA
37	DI	2	V4	Miseq1	2	12+18	HF	MB	515 & 806rcb
38	5NDI	2	V4	Miseq1	2	12+18	HF	MB	515 & 806rcbc27
39	FG	3	V4	Miseq2	10	15	HF	MB	515 & 806rcbc27
40	FG	3	V4	Miseq2	10	15	HF	MB	515 & 806rcbc28
41	FG	3	V4	Miseq2	10	15	HF	MB	515 & 806rcbc29
42	FG	3	V4	Miseq2	1	25	HF	MB	515 & 806rcbc30
43	FG	3	V4	Miseq2	1	25	HF	MB	515 & 806rcbc31
44	FG	3	V4	Miseq2	1	25	HF	MB	515 & 806rcbc32
45	FG	3	V4	Miseq2	10	25	HF	MB	515 & 806rcbc33

Table S4: Overview of experimental design for data sets (2).

Meta ID	Lib. Prep.	Run	Region	Machine	input ng	PCR cycle (R1+R2)	Taq	Template	F & R primer
46	FG	3	V4	Miseq2	10	25	HF	MB	515 & 806rcbc34
47	FG	3	V4	Miseq2	10	25	HF	MUB	515 & 806rcbc35
48	DI	4	V4	Miseq2	2	12+20	HF	MUB	F515A & 805RA
49	DI	4	V4	Miseq2	2	12+20	HF	MUB	F515A & 805RA
50	XT	4	16S	Miseq2	2	20	HF	MB	27YMF & 1492R
51	XT	4	16S	Miseq2	2	20	HF	MUB	27YMF & 1492R
52	DI	5	V4	Miseq2	2	5+15	HF	MB	F515A & 805RA
53	DI	5	V4	Miseq2	2	8+15	HF	MB	F515A & 805RA
54	DI	5	V4	Miseq2	2	10+15	HF	MB	F515A & 805RA
59	DI	5	V4	Miseq2	2	8+15	HF	MB	F515A & 805RA
60	DI	5	V4	Miseq2	2	10+15	HF	MB	F515A & 805RA
61	DI	5	V4	Miseq2	2	10+15	HF	MB	F515A & 805RA
62	DI	5	V4	Miseq2	2	8+15	HF	MUB	F515A & 805RA
64	DI	5	V4	Miseq2	2	8+15	HF	MUB	F515A & 805RA
65	DI	5	V4	Miseq2	2	10+15	HF	MUB	F515A & 805RA
66	DI	5	V4	Miseq2	2	10+15	HF	MUB	F515A & 805RA
67	DI	5	V4	Miseq2	2	10+15	HF	MUB	F515A & 805RA
68	DI	5	V4	Miseq2	2	8+15	HF	HA	F515A & 805RA
69	DI	5	V4	Miseq2	2	8+15	HF	LC	F515A & 805RA
71	DI	5	V4	Miseq2	2	8+15	HF	RBS	F515A & 805RA
74	DI	5	V4	Miseq2	2	8+15	Q5	MB	F515A & 805RA
75	DI	5	V4	Miseq2	2	8+15	Q5	MB	F515A & 805RA
76	DI	5	V4	Miseq2	2	8+15	Q5	MB	F515A & 805RA
77	FG	6	V4	Miseq1	2	15	HF	MUB	515 & 806rcbc5
78	FG	6	V4	Miseq1	5	15	Q5	MB	515 & 806rcbc8
79	FG	6	V4	Miseq1	5	15	Q5	MB	515 & 806rcbc9
80	FG	6	V4	Miseq1	5	25	Q5	MB	515 & 806rcbc0
81	FG	6	V4	Miseq1	5	25	Q5	MB	515 & 806rcbc1
82	FG	6	V4	Miseq1	5	15	HF	MB	515 & 806rcbc2
83	FG	6	V4	Miseq1	5	15	HF	MB	515 & 806rcbc3
85	FG	6	V4	Miseq1	5	25	HF	MB	515 & 806rcbc5
86	DI	7	V4	Miseq2	2	10+15	HF	MB	515 & 806rcb
87	DI	7	V4	Miseq2	2	10+15	HF	MB	515 & 806rcb
88	DI	7	V4	Miseq2	2	10+15	HF	MB	515 & 806rcb
89	DI	7	V4	Miseq2	2	10+15	HF	MUB	515 & 806rcb
90	DI	7	V4	Miseq2	2	10+15	HF	MUB	515 & 806rcb
91	DI	7	V4	Miseq2	2	10+15	HF	MUB	515 & 806rcb
93	DI	7	V4	Miseq2	2	10+15	HF	AT	515 & 806rcb
94	DI	7	V4	Miseq2	2	10+15	HF	BT	515 & 806rcb
96	DI	7	V4	Miseq2	2	10+15	HF	CS	515 & 806rcb
97	DI	7	V3/V4	Miseq2	2	10+15	HF	MB	341f & 806rcb
98	DI	7	V3/V4	Miseq2	2	10+15	HF	MB	341f & 806rcb
99	DI	7	V3/V4	Miseq2	2	10+15	HF	MB	341f & 806rcb
100	DI	7	V3/V4	Miseq2	2	10+15	HF	MB	341f & 805RA
101	DI	7	V3/V4	Miseq2	2	10+15	HF	MB	341f & 805RA
102	DI	7	V3/V4	Miseq2	2	10+15	HF	MB	341f & 805RA

Availability of data sets:

Table S5: Overview of accession numbers and corresponding Meta ID for all data sets.

Sample accession	Secondary accession	Meta ID	Sample accession	Secondary accession	Meta ID
ERS447105	SAMEA2494039	19	ERS447148	SAMEA2494082	62
ERS447106	SAMEA2494040	20	ERS447149	SAMEA2494083	63
ERS447107	SAMEA2494041	21	ERS447150	SAMEA2494084	64
ERS447108	SAMEA2494042	22	ERS447151	SAMEA2494085	65
ERS447109	SAMEA2494043	23	ERS447152	SAMEA2494086	66
ERS447110	SAMEA2494044	24	ERS447153	SAMEA2494087	67
ERS447111	SAMEA2494045	25	ERS447154	SAMEA2494088	68
ERS447112	SAMEA2494046	26	ERS447155	SAMEA2494089	69
ERS447113	SAMEA2494047	27	ERS447156	SAMEA2494090	70
ERS447114	SAMEA2494048	28	ERS447157	SAMEA2494091	71
ERS447115	SAMEA2494049	29	ERS447158	SAMEA2494092	72
ERS447116	SAMEA2494050	30	ERS447159	SAMEA2494093	73
ERS447117	SAMEA2494051	31	ERS447160	SAMEA2494094	74
ERS447118	SAMEA2494052	32	ERS447161	SAMEA2494095	75
ERS447119	SAMEA2494053	33	ERS447162	SAMEA2494096	76
ERS447120	SAMEA2494054	34	ERS447163	SAMEA2494097	77
ERS447121	SAMEA2494055	35	ERS447164	SAMEA2494098	78
ERS447122	SAMEA2494056	36	ERS447165	SAMEA2494099	79
ERS447123	SAMEA2494057	37	ERS447166	SAMEA2494100	80
ERS447124	SAMEA2494058	38	ERS447167	SAMEA2494101	81
ERS447125	SAMEA2494059	39	ERS447168	SAMEA2494102	82
ERS447126	SAMEA2494060	40	ERS447169	SAMEA2494103	83
ERS447127	SAMEA2494061	41	ERS447170	SAMEA2494104	84
ERS447128	SAMEA2494062	42	ERS447171	SAMEA2494105	85
ERS447129	SAMEA2494063	43	ERS447172	SAMEA2494106	86
ERS447130	SAMEA2494064	44	ERS447173	SAMEA2494107	87
ERS447131	SAMEA2494065	45	ERS447174	SAMEA2494108	88
ERS447132	SAMEA2494066	46	ERS447175	SAMEA2494109	89
ERS447133	SAMEA2494067	47	ERS447176	SAMEA2494110	90
ERS447134	SAMEA2494068	48	ERS447177	SAMEA2494111	91
ERS447135	SAMEA2494069	49	ERS447178	SAMEA2494112	92
ERS447136	SAMEA2494070	50	ERS447179	SAMEA2494113	93
ERS447137	SAMEA2494071	51	ERS447180	SAMEA2494114	94
ERS447138	SAMEA2494072	52	ERS447181	SAMEA2494115	95
ERS447139	SAMEA2494073	53	ERS447182	SAMEA2494116	96
ERS447140	SAMEA2494074	54	ERS447183	SAMEA2494117	97
ERS447141	SAMEA2494075	55	ERS447184	SAMEA2494118	98
ERS447142	SAMEA2494076	56	ERS447185	SAMEA2494119	99
ERS447143	SAMEA2494077	57	ERS447186	SAMEA2494120	100
ERS447144	SAMEA2494078	58	ERS447187	SAMEA2494121	101
ERS447145	SAMEA2494079	59	ERS447188	SAMEA2494122	102
ERS447146	SAMEA2494080	60			
ERS447147	SAMEA2494081	61			

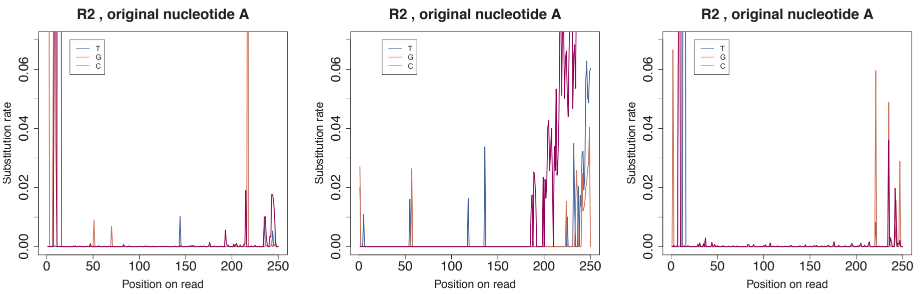

(a) Sub. profiles for R2 (orig. nucleotide A): *Bacteroides thetaiotaomicron VPI-5482*

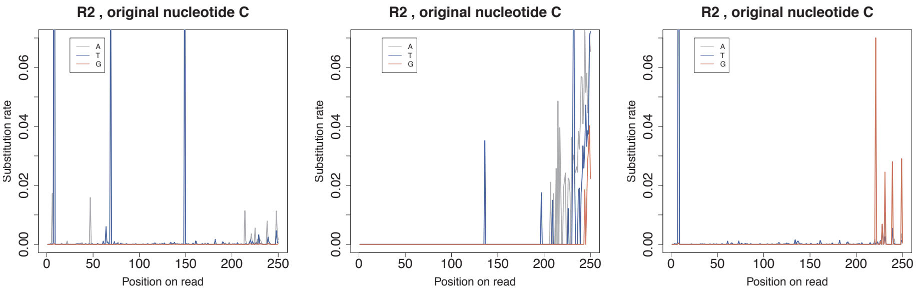

(b) Sub. profiles for R2 (orig. nucleotide C): *Bacteroides thetaiotaomicron VPI-5482*

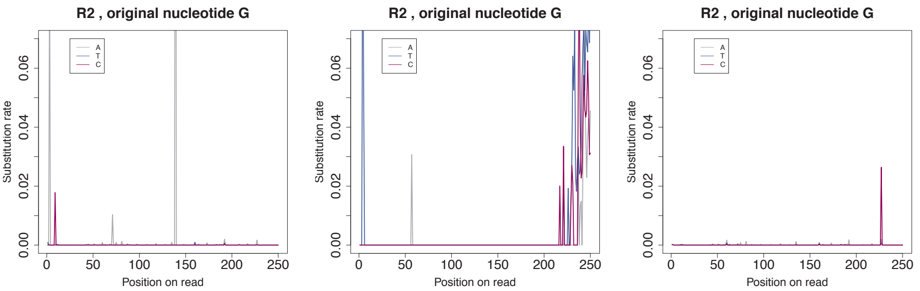

(c) Sub. profiles for R2 (orig. nucleotide G): *Bacteroides thetaiotaomicron VPI-5482*

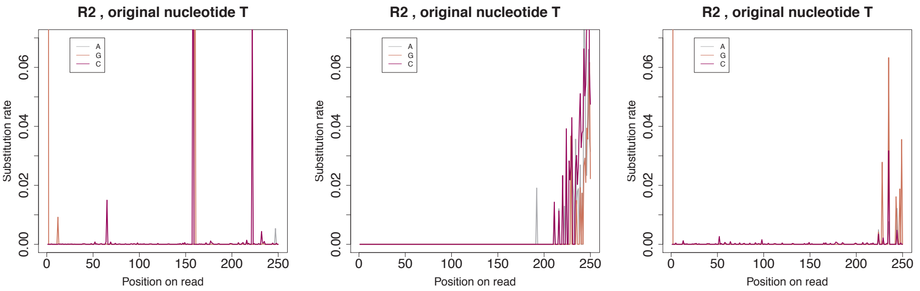

(d) Sub. profiles for R2 (orig. nucleotide T): *Bacteroides thetaiotaomicron VPI-5482*

Figure S2: The figure displays the error profiles for three data sets where the organism *Bacteroides thetaiotaomicron VPI-5482* was sequenced. Each library was constructed with a different method. For the data set displayed in the first column the nested single index was used, for the data set displayed in the second column NexteraXT was used and the last library was constructed with the nested dual index.

Table S6: Overview of organisms in the mock community.

**Bacteria**	
Acidobacterium capsulatum ATCC 51196	*Ruegeria pomeroyi DSS-3*
Akkermansia muciniphila ATCC BAA-835	*Salinispora arenicola CNS-205*
Anaerocellum thermophilum Z-1320, DSM 6725	*Salinispora tropica CNB-440*
Bacteroides thetaiotaomicron VPI-5482	*Shewanella baltica OS185*
Bacteroides vulgatus ATCC 8482	*Shewanella baltica OS223*
Bordetella bronchiseptica RB50	*Sulfitobacter sp. EE-36*
Burkholderia xenovorans LB400	*Sulfitobacter sp. NAS-14.1*
Caldicellulosiruptor saccharolyticus DSM 8903	*Sulfurihydrogenibium sp. YO3AOP1*
Chlorobaculum tepidum TLS	*Sulfurihydrogenibium yellowstonense SS-5*
Chlorobium limicola DSM 245	*Thermoanaerobacter pseudethanolicus ATCC 33223*
Chlorobium phaeobacteroides DSM 266	*Thermotoga neapolitana DSM 4359*
Chlorobium phaeovibrioides DSM 265	*Thermotoga petrophila RKU-1*
Chloroflexus aurantiacus J-10-fl	*Thermotoga sp. RQ2*
Clostridium thermocellum ATCC 27405	*Thermus thermophilus HB8*
Deinococcus radiodurans R1	*Treponema denticola ATCC 35405*
Desulfovibrio desulfuricans desulfuricans ATCC 27774	*Treponema vincentii I*
Desulfovibrio piger ATCC 29098	*Zymomonas mobilis mobilis ZM4*
Dictyoglomus turgidum DSM 6724	
Erwinia chrysanthemi	
Enterococcus faecalis V583v	
Fusobacterium nucleatum nucleatum ATCC 25586	
Gemmatimonas aurantiaca T-27T	**Archaea**
Herpetosiphon aurantiacus ATCC 23779	*Archaeoglobus fulgidus DSM 4304*
Hydrogenobaculum sp. Y04AAS1	*Ignicoccus hospitalis KIN4/I*
Leptothrix cholodnii SP-6	*Methanocaldococcus jannaschii DSM 2661*
Nitrosomonas europaea ATCC 19718	*Methanococcus maripaludis C5*
Nostoc sp. PCC 7120	*Methanococcus maripaludis S2*
Pelodictyon phaeoclathratiforme BU-1	*Nanoarchaeum equitans Kin4-M*
Persephonella marina EX-H1	*Pyrobaculum aerophilum IM2*
Porphyromonas gingivalis ATCC 33277	*Pyrobaculum calidifontis JCM 11548*
Rhodopirellula baltica SH 1	*Pyrococcus horikoshii OT3*
Rhodospirillum rubrum ATCC 11170	*Sulfolobus tokodaii 7(S311)*

Permutation ANOVA for Error Profiles

We used the Hellinger distance to create distance matrices followed by a permutation ANOVA to determine how much of the variation can be explained by the environmental factors and to identify the factors driving the clustering that we observed in Figure 5.

Environmental factors: library preparation method, run, region, machine, input ng, PCR Cycle (R1+R2), taq, template, forward+reverse primer

R1 + R2 Substitutions

We used stepwise regression (R function: step()) with Redundancy Analysis (RDA) (R function: rda()) to determine which factors to include and their order based on the R1 data and used the same model for R2. The resulting model (library preparation method + run + input ng + PCR cycle R1&R2 + taq + template + forward & reverse primer) was used for the adonis() function of the vegan package.

Table S7: Results for R1 substitutions.

	Df	SumsOfSqs	MeanSqs	F.Model	R2	Pr(>F)
Library Preparation Method	3	632.26	210.753	24.1463	0.36841	0.001 ***
Run	1	110.02	110.018	12.6049	0.06411	0.001 ***
input ng	1	19.29	19.286	2.2096	0.01124	0.040 *
PCR Cycle R1+R2	9	234.16	26.018	2.9809	0.13644	0.001 ***
Taq	1	31.02	31.025	3.5546	0.01808	0.004 **
Template	8	122.45	15.307	1.7537	0.07135	0.002 **
F R Primer	13	340.06	26.158	2.9970	0.19815	0.001 ***
Residuals	26	226.93	8.728		0.13223	
Total	62	1716.19			1.00000	

Table S8: Results for R2 substitutions.

	Df	SumsOfSqs	MeanSqs	F.Model	R2	Pr(>F)
Library Preparation Method	4	1017.07	254.267	20.5682	0.44486	0.001 ***
Run	1	112.37	112.374	9.0902	0.04915	0.001 ***
input ng	1	60.47	60.469	4.8915	0.02645	0.001 ***
PCR Cycle R1 + R2	10	310.93	31.093	2.5151	0.13600	0.001 ***
Taq	1	18.37	18.374	1.4863	0.00804	0.129
Template	8	180.77	22.597	1.8279	0.07907	0.001 ***
F R PRIMER	5	165.95	33.190	2.6848	0.07259	0.001 ***
Residuals	34	420.31	12.362		0.18384	
Total	64	2286.24			1.00000	

Multidimensional scaling (MDS) plots for quality profiles of insertions and deletions

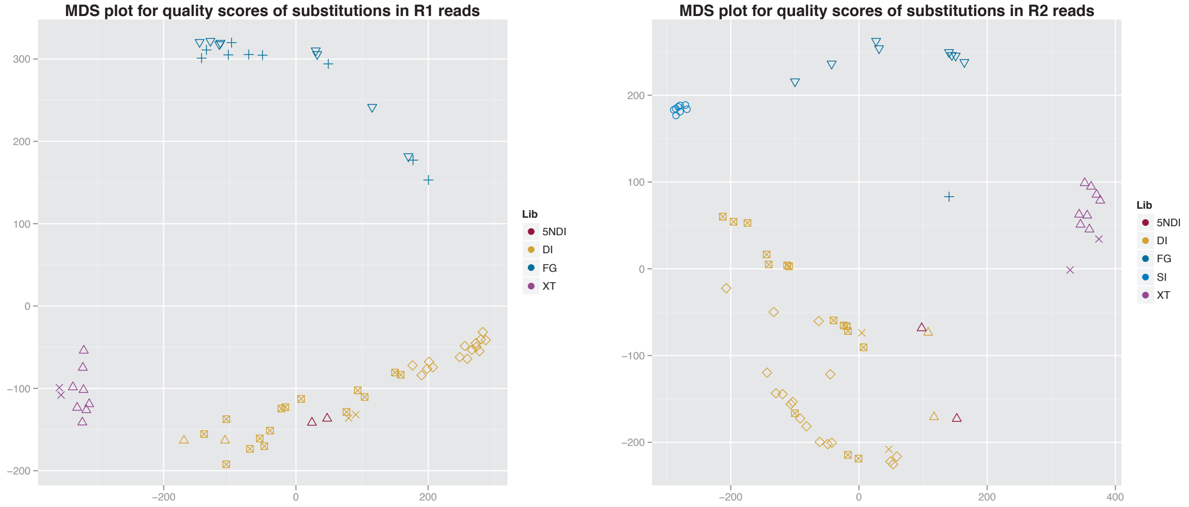

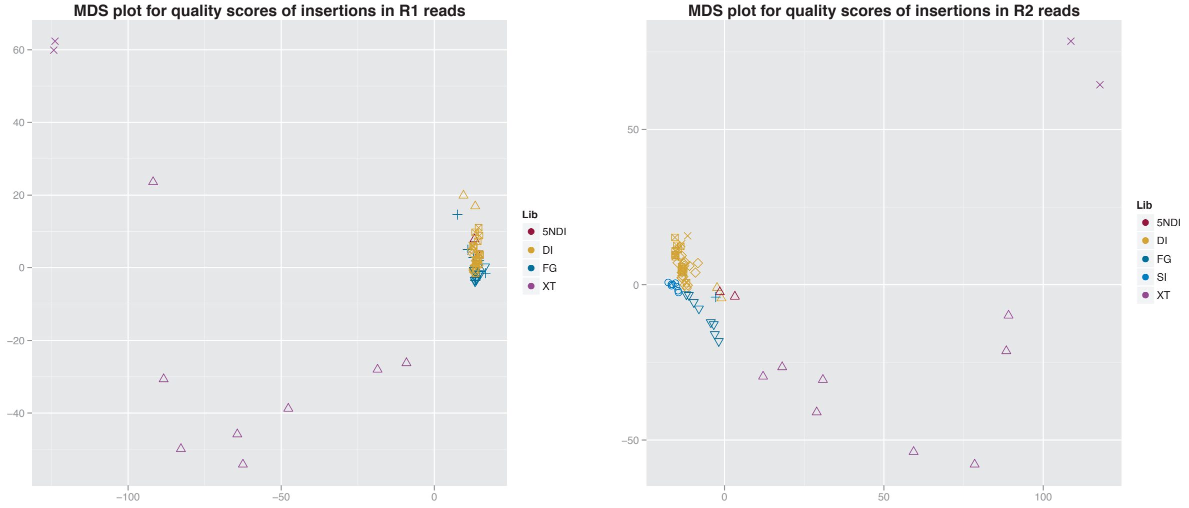

(b) Insertions

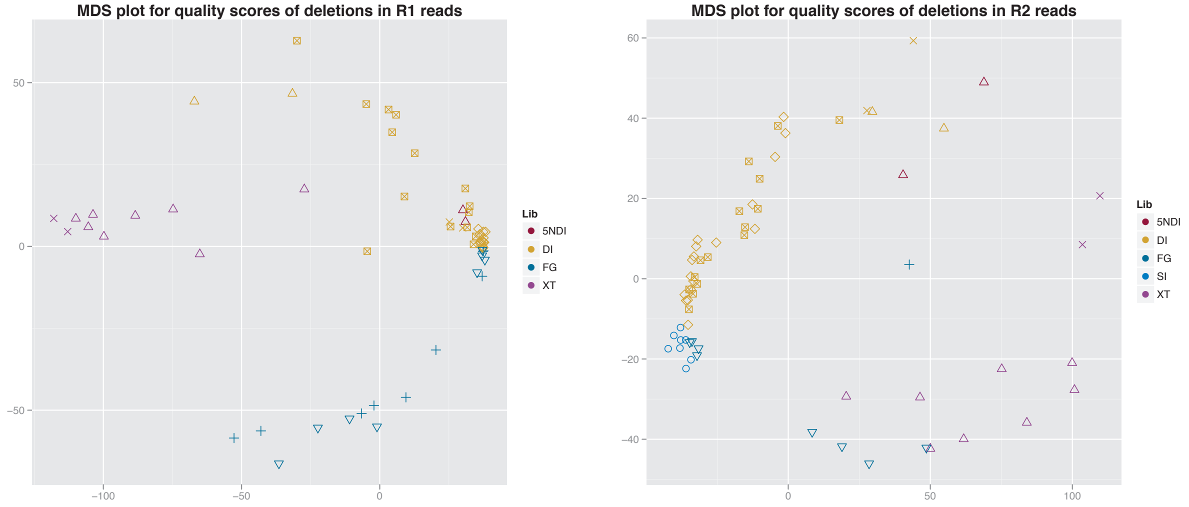

(c) Deletions

Figure S3: We used to Hellinger distance to construct a similarity matrix for the position specific quality distributions. The different library preparations methos of the data sets are indicated by colour and the run information is identified by shape.

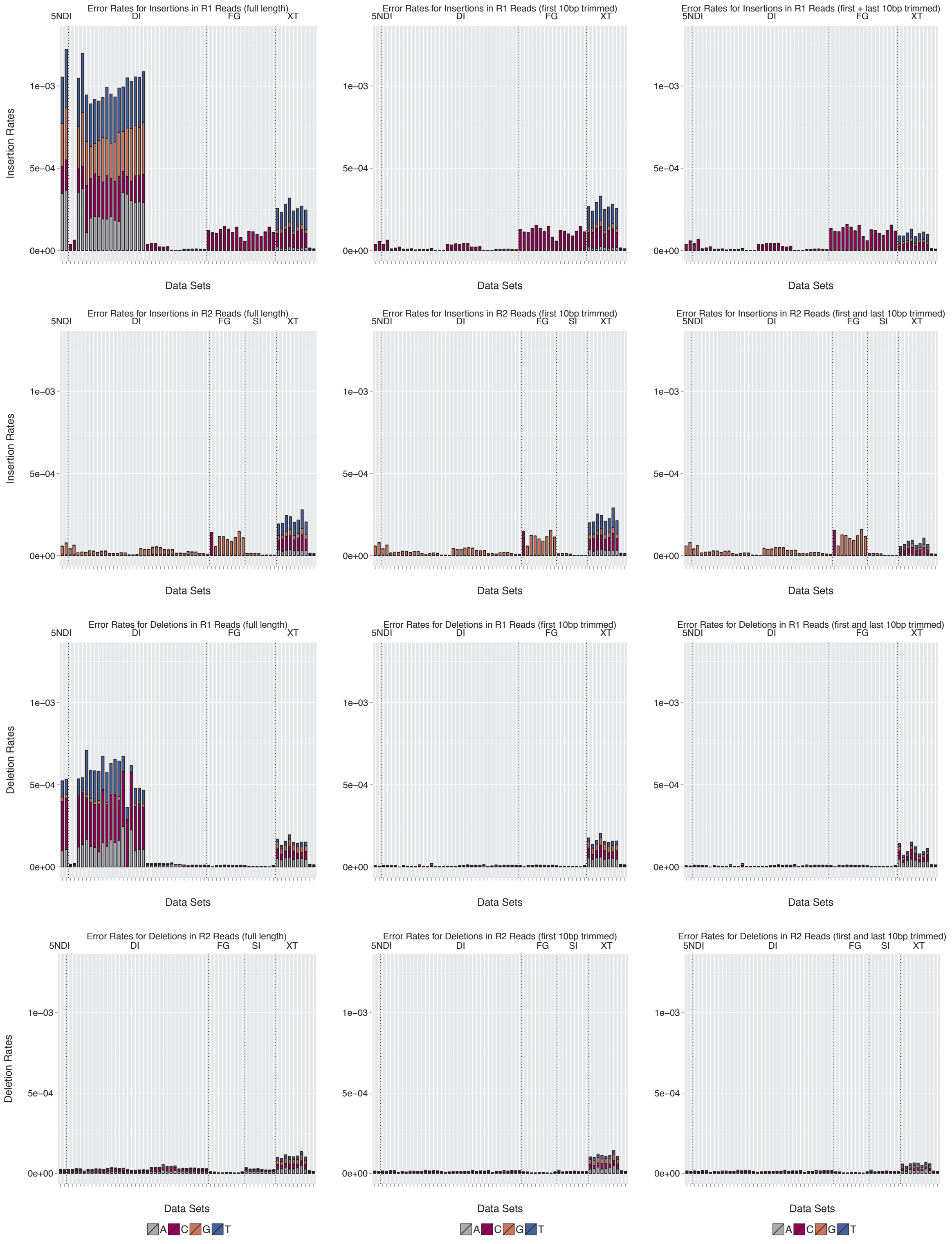

Figure S4: Trimming the start and end of the read to remove indels: The first column shows the R1 and R2 indel rates for the raw reads (full length). The second column shows the error rates after trimming the first 10bp and the last column shows the error rates after additionally trimming the last 10bp. Data sets indicated on the x axis grouped by library preparation method (from left to right): 36, 38, 35, 37, 48, 49, 54, 59, 60, 61, 62, 64, 65, 66, 67, 68, 69, 71, 74, 75, 76, 86, 87, 88, 89, 90, 91, 93, 94, 96, 97, 98, 99, 100, 101, 102, 39, 40, 41, 42, 43, 44, 45, 46, 47, 77, 78, 79, 80, 81, 82, 83, 85, 27, 28, 29, 30, 31, 32, 33, 34, 50, 51

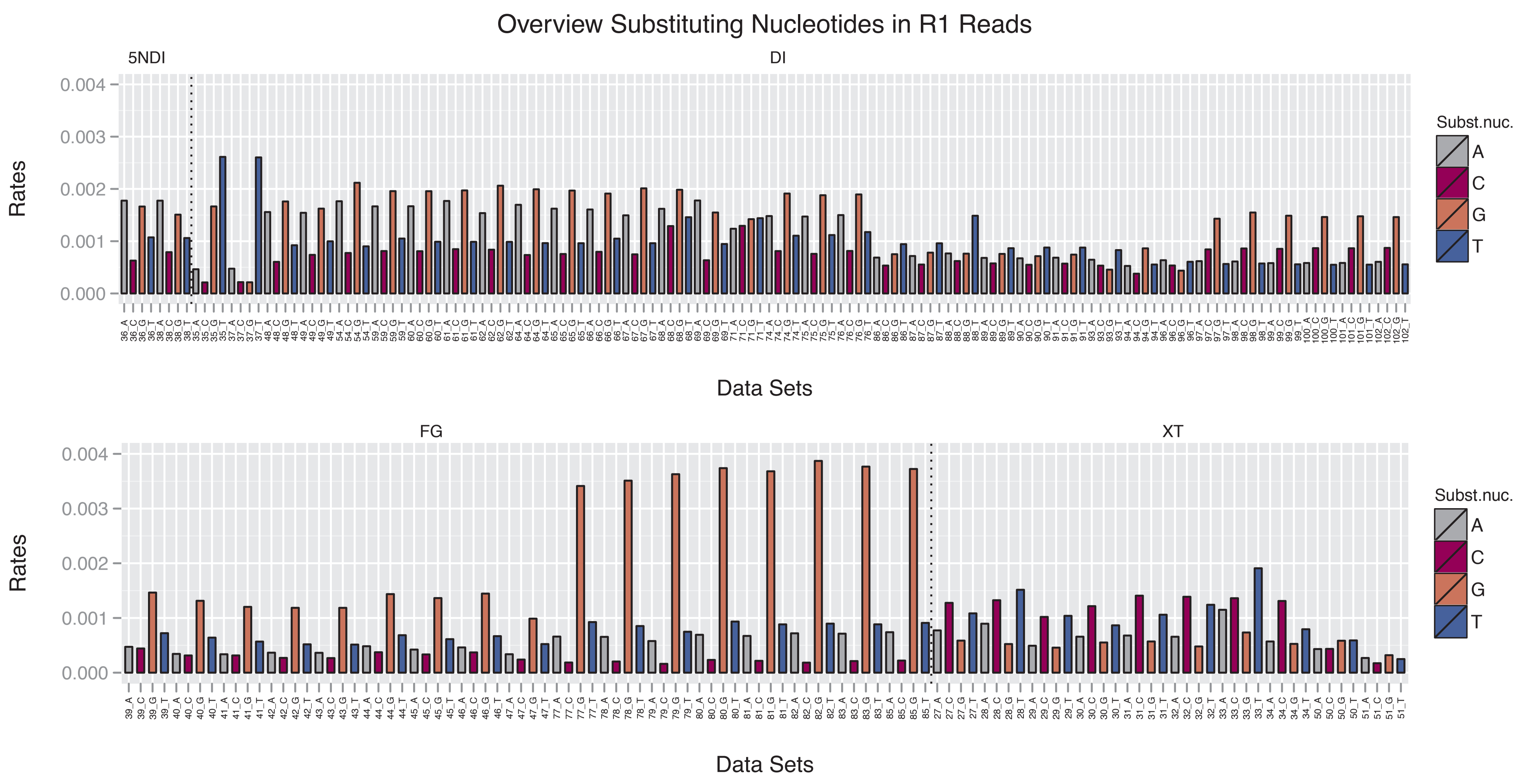

Figure S5: The figure indicates the rate of each substituting nucleotide in R1 reads for the 5N dual index and dual index data sets (upper plot) and the Fusion Golay and NexteraXT data sets (lower plot).

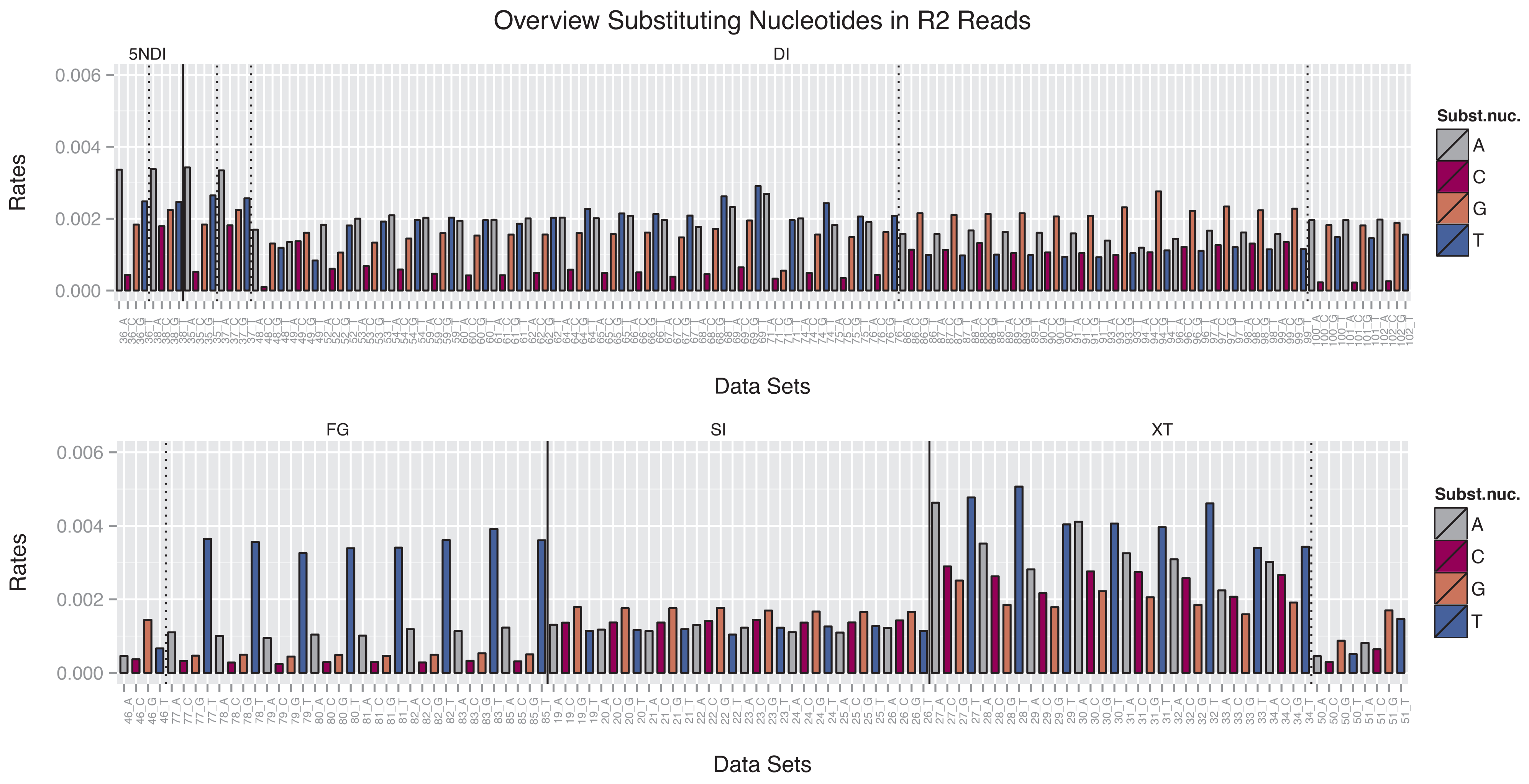

Figure S6: The figure indicates the rate of each substituting nucleotide in R2 reads for the 5N dual index and dual index data sets (upper plot) and the Fusion Golay, single index and NexteraXT data sets (lower plot).

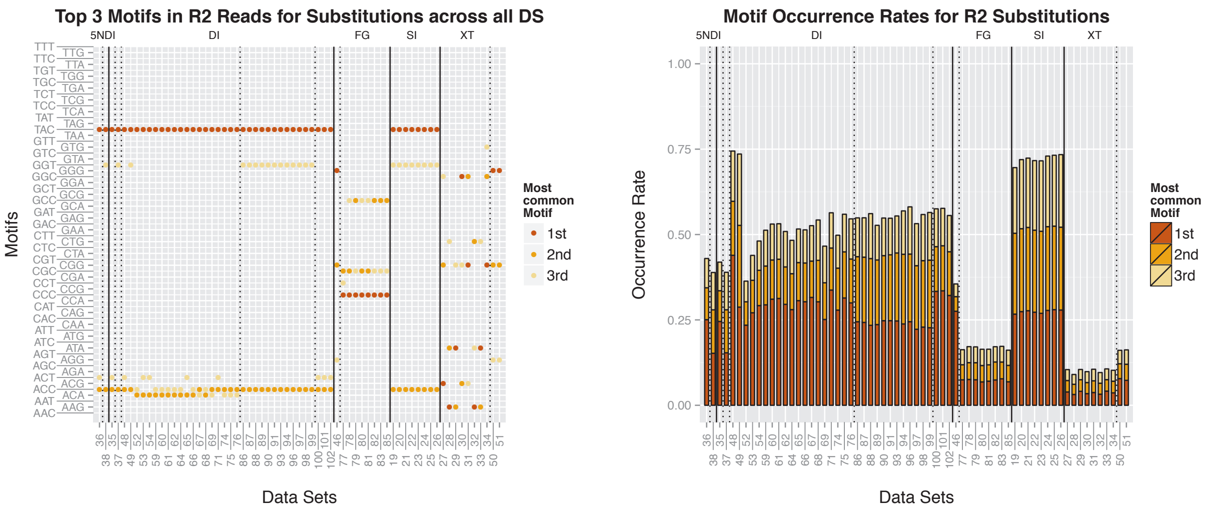

(a) R2 substitutions

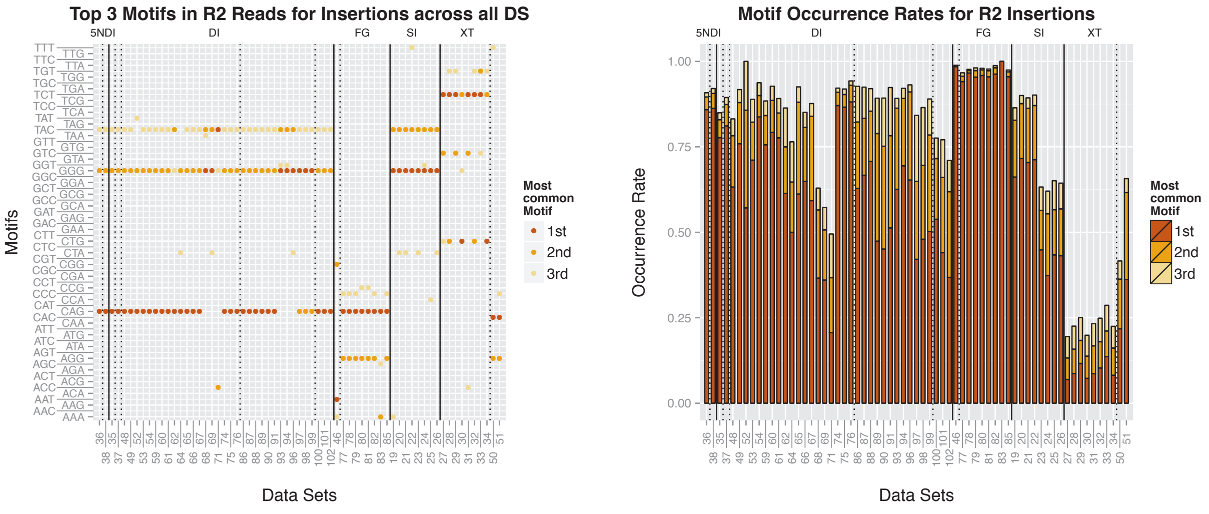

(b) R2 insertions

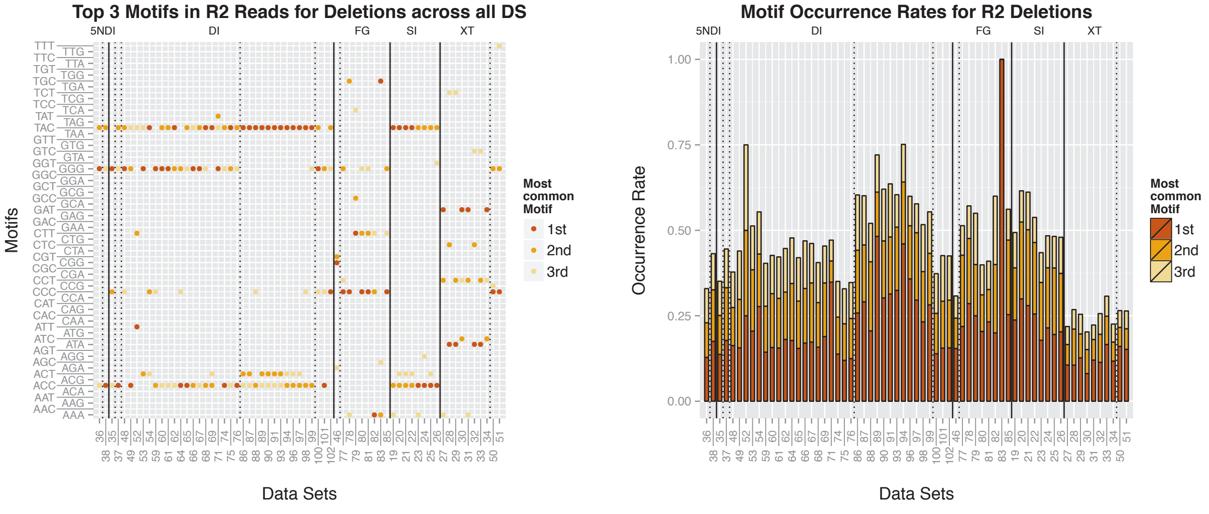

(c) R2 deletions

Figure S7: We recorded all 3mers preceding a substitution, insertion or deletion error in R2 reads, respectively. The first column displays the 3 most common motifs for each data set and the second column illustrates the percentage of errors that were associated with the respective motif. Data sets are grouped by library preparation (solid lines) and primers (dotted lines).

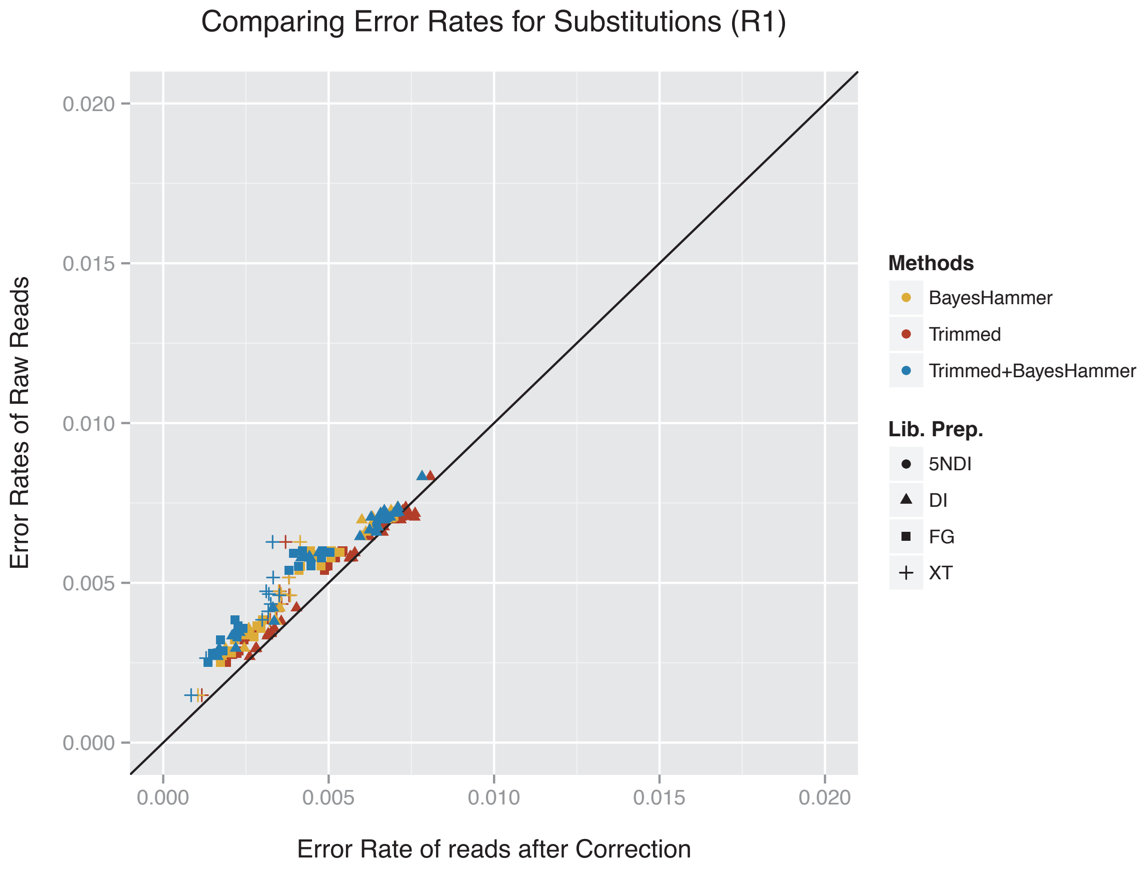

Figure S8: The figure displays the R1 results for different error correction approaches (keeping R1 and R2 reads separate). First trimming the reads and then applying BayesHammer yields slightly better results than each method on its own. We included only the data sets in the figure for which we had at least 1,000 aligned reads for all methods. Excluded data sets: 19-26 (no results on raw reads), 52 (no results across all methods), 53 (no results on raw reads).

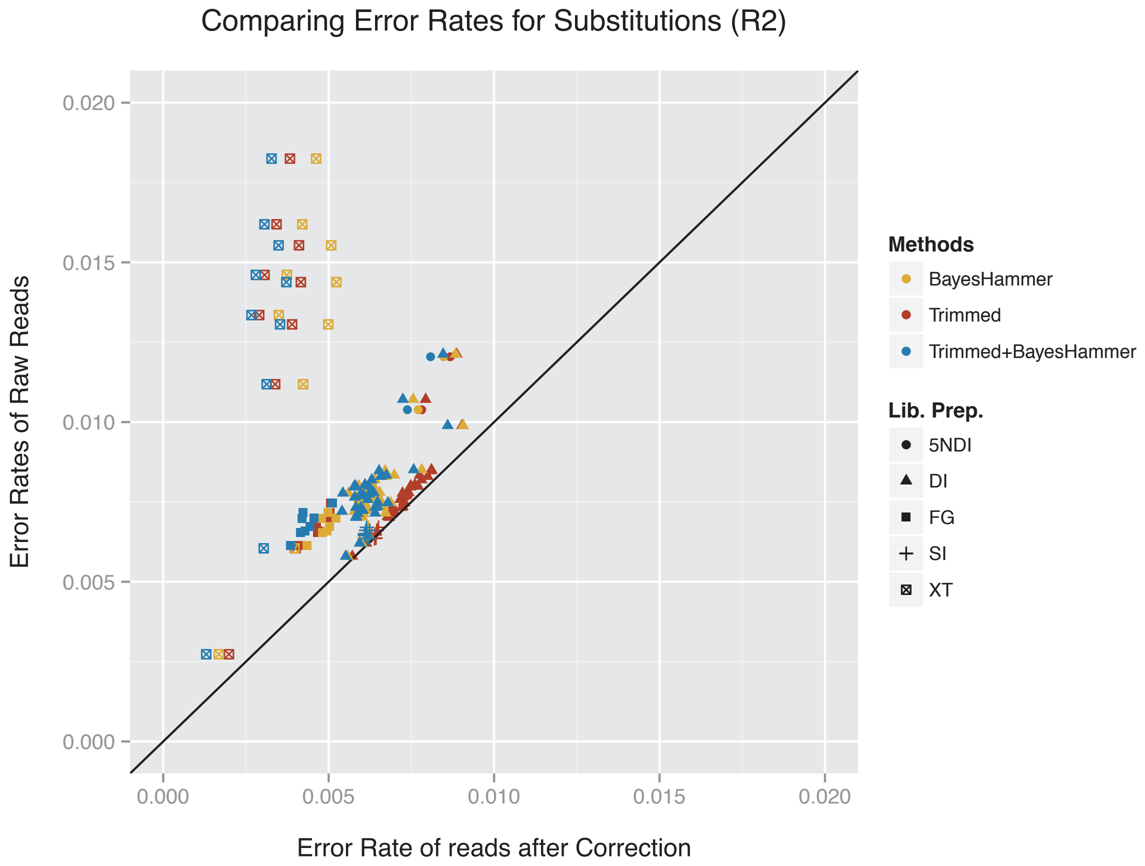

Figure S9: The figure displays the R2 results analogously (see figure S8). Excluded data sets: 39-41+43 (no results across all methods), 42+44+45+47 (no results for raw reads, BayesHammer, trimming+BayesHammer), 46+52 (no results for trimming+BayesHammer).

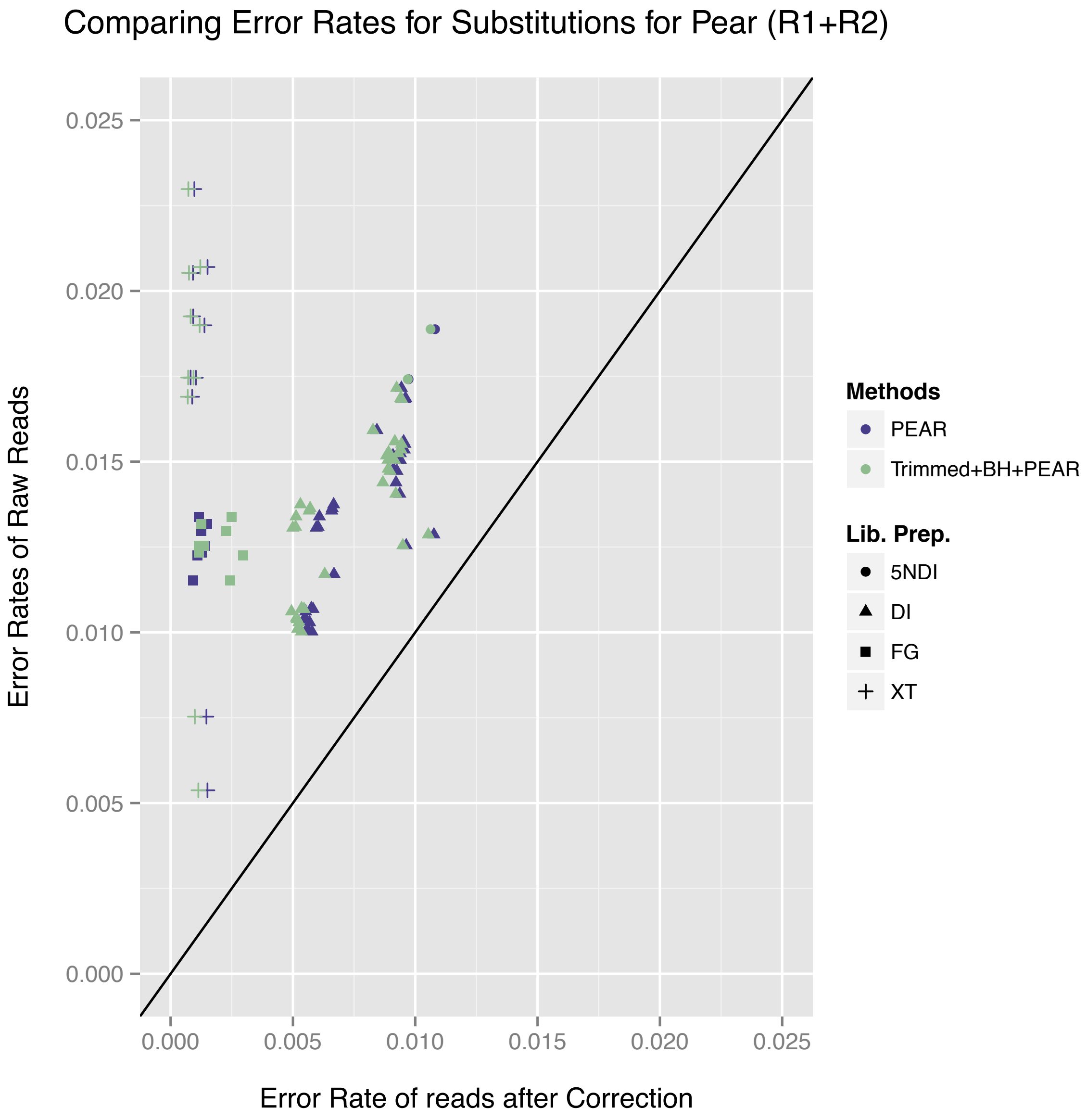

Figure S10: The figure summarises the results for directly applying PEAR to the raw reads and after processing the reads with Sickle and BayesHammer. Excluded data sets: 19-26, 39-45, 47 (not enough aligned raw reads), 46 (no results for PEAR), 52 (no results across all methods), 53 (no results for raw reads and PEAR).

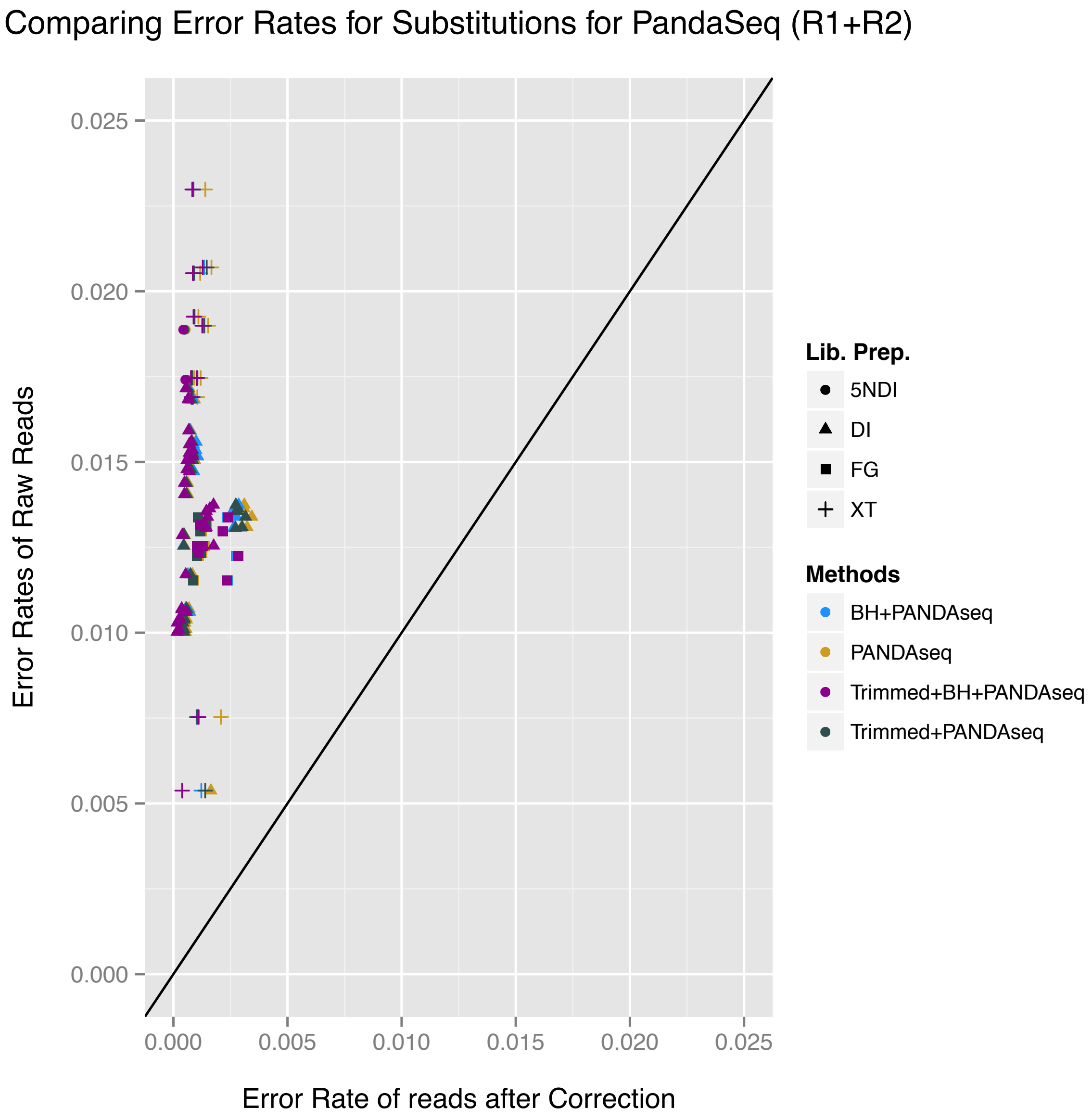

Figure S11: The figure summarises the results for PANDAseq combined with trimming and BayesHammer. (The 50bp overlap was reduced to 10bp for the V3/V4 data sets for Trimmed+BH+PANDAseq.) Excluded data sets: For raw reads: 19-26, 39-45, 47,52, 53; for PANDAseq: 20, 24, 25, 46; for BH+PANDAseq: 46; for trimming+PANDAseq: none; for trimming+BH+PANDAseq: none
